# Activities and participation of children with language disorders in outpatient care according to the ICF

**DOI:** 10.1590/2317-1782/20232022007en

**Published:** 2023-08-04

**Authors:** Gabriela Damaris Ribeiro Nogueira, Stela Maris Aguiar Lemos, Denise Brandão de Oliveira e Britto

**Affiliations:** 1 Departamento de Fonoaudiologia, Faculdade de Medicina, Universidade Federal de Minas Gerais - UFMG - Belo Horizonte (MG), Brasil.

**Keywords:** Child Language, Language Development, Language Development Disorders, Child, International Classification of Functioning, Disability and Health

## Abstract

**Purpose:**

To identify the main categories of the Activities and Participation component of the International Classification of Functioning, Disability, and Health and to verify the association with age, gender, education, and speech therapy diagnosis in children who are assisted by an oral language clinic.

**Methods:**

This is an analytical and cross-sectional observational study, carried out with secondary data from 32 medical records of children with the majority male, mean age of 41.03 months, in early childhood education and language disorder associated with other conditions. The main speech-language pathology manifestations were coded according to the pre-selected categories of the Activities and Participation component, and descriptive and bivariate statistical analyzes were performed, and the Fisher's Exact test was used with a significance level of 5%.

**Results:**

The descriptive analysis of the pre-selected categories allowed us to verify a high number of “Not informed” answers, with a higher percentage in vocal expression without speech (d331) performance (93.8%), making decisions (d177) ability (90.6%), problem solving (d175) performance (65.6%) and capacity (87.5%), reception of oral messages (d310) performance (65.6%) and eating (d550) capacity (65.6%). Among the 24 categories selected, 12 jointly contemplated the Qualifiers of Performance and Capacity. There was a statistically significant association between the three categories with sociodemographic data and speech therapy diagnoses.

**Conclusion:**

Difficulties were identified in several categories of the Activities and Participation component and statistically significant associations between them and sociodemographic data and speech therapy diagnoses, showing the impacts of oral language disorders on the activities and participation of children assisted in an outpatient speech therapy service.

## INTRODUCTION

Language is a communication system essential to human functioning, through which people interact with society. Acquiring and refining speech and language skills throughout child development enable them to have greater control of their lives as they express their needs, feelings, and ideas. Child language impairments can impair or have an impact on their psychosocial and cognitive development and quality of life^(1-3)^.

Child language assessment and therapeutic strategy planning in rehabilitation should address not only organic aspects but also the influence of environmental, psychosocial, and cognitive aspects. Such an approach provides a wider understanding of language, encompassing its use in the various contexts of life and how it shapes children’s functioning^(4)^.

The International Classification of Functioning, Disability, and Health (ICF) belongs to the family of international classifications of the World Health Organization (WHO), whose purpose is to describe health and related statuses and provide a unified and standardized language^(5)^. Hence, the tool classifies functioning, disability, and contextual factors associated with health conditions from a biopsychosocial perspective^(5)^.

Using ICF to monitor the progress of functioning helps identify, understand, and monitor the impacts language impairment may have on children’s overall and social development, besides favoring assistance based on comprehensive health attention and care and related statuses. As it helps identify aspects of functioning, especially the ones related to activity limitations and participation restrictions, ICF characterization can help plan interventions to overcome barriers in different life contexts^(2)^.

The American Speech-Language-Hearing Association (ASHA) recommends incorporating ICF in all healthcare components to integrate the influence of contextual factors of functioning and promote the development of functional objectives and collaborative practices in clinical decisions^(6)^.

Given its complexity and size and the lack of studies and reference tools, ICF use in speech-language-hearing (SLH) practice must increase by developing standardized clinical instruments to guide and help its clinical application^(7)^. There must also be studies associating categories relevant to oral language changes with personal factors and clinical diagnoses, thus helping understand language impairment in greater depth and consider its individual and social consequences.

ICF is structured in two parts. The first one, Functioning and Disability, includes the components of Body Functions and Structures, and Activities and Participation. The second part addresses information on the context, including the components of Environmental Factors and Personal Factors. Positive aspects (functional and structural integrity, activities and participation) are named Functioning. Negative aspects (disabilities, activity limitations, or participation restrictions) make up Disability. Concerning environmental factors, positive aspects are the facilitators, and negative ones are the barriers^(5)^.

This study focused on Activities and Participation, a component that encompasses the qualifiers of Capacity and Performance and includes domains that indicate individual and social aspects of functioning; hence, it represents the ICF “information matrix”^(5)^. Language changes in children can hinder them from carrying out tasks and actions (Activities) and getting involved in daily life situations (Participation), respectively causing limitations and restrictions. Therefore, it is greatly important to investigate changes in Language and Functioning.

Thus, this study aimed to identify the main categories of ICF Activities and Participation and the possible Capacity and Performance qualifiers and verify their associations with age, sex, educational attainment, and overall SLH diagnosis in children in oral language outpatient care.

## METHODS

This research was approved by the Research Ethics Committee of the Federal University of Minas Gerais under number 3.172.707 and CAAE: 02470618.1.0000.5149.

This cross-sectional analytical observational study analyzed secondary data collected from the medical records of patients attending an SLH outpatient service at a public hospital.

The service in question is a pediatric oral language therapy outpatient center that treats children with language disorders associated with Down syndrome and other genetic or sensory conditions; autism spectrum disorder; developmental language disorder, and speech sound disorder.

The sample comprised the data collected from the medical records of 32 patients - i.e., all those who attended the service between August 2018 and March 2021. They were 14 months to 8 years old and had been diagnosed with pediatric oral language disorder. All research participants’ parents/guardians signed an informed consent form and authorized the access to the participants’ medical records.

Firstly, the following data were collected from the initial assessment reports: sex, date of birth, educational attainment, and overall SLH diagnosis. Then, the main SLH manifestations and diagnoses were classified according to the ICG Activities and Participation, using its Capacity and Performance qualifiers.

The survey considered the SLH manifestations and suspected diagnoses related to language changes in its receptive and expressive aspects; the phonological, morphosyntactic, pragmatic, and semantic subsystems; and the cognitive development aspects described in the reports based on the results of the following tests: Behavioral Observation Protocol (PROC, in Portuguese)^(8)^, Language Development Assessment (ADL)^(9)^, Behavior Observation Protocol for children up to 6 years old^(10)^, ABFW Child Language Test^(11)^, USP Picture Vocabulary Test (TVfusp)^(12)^, Auditory Vocabulary Test (TVAud), and Expressive Vocabulary Test (TVExp)^(13)^. Hearing assessment results, when present, were also considered.

ICF categories were defined by preselecting Activities and Participation chapters that were related to pediatric oral language changes. The preselection was open - i.e., considering the SLH manifestations and diagnoses listed in the reports. This study included five chapters: “Learning and applying knowledge”, “General tasks and demands”, “Communication”, “Self-care”, and “Major life areas” (Chart 1).

**Chart 1:** Categories selected from the ICF Activities and Participation component

Chapters	Categories
Chapter 1. Learning and applying knowledge	d110 - Watching
	d115 - Listening
	d130 - Copying
	d131 - Learning through actions with objects
	d132 - Acquiring language
	d137 - Acquiring concepts
	d155 - Acquiring skills
	d160 - Focusing attention
	d175 - Solving problems
	d177 - Making decisions
Chapter 2. General tasks and demands	d230 - Carrying out daily routine
	d240 - Handling stress and other psychological demands
Chapter 3. Communication	d310 - Communicating with - receiving - spoken messages
	d330 - Speaking
	d331 - Non-speech vocal expression
	d335 - Producing nonverbal messages
	d350 - Conversation
Chapter 5. Self-care	d510 - Washing oneself
	d520 - Caring for body parts
	d540 - Dressing
	d550 - Eating
Chapter 8. Major life areas	d815 - Preschool education
	d820 - School education

After defining the chapters, their set of categories was selected, and then associating their Performance and Capacity qualifier codes with the report findings. This study used only the following qualifiers: .0 (no difficulties), .8 (not specified difficulty), and .9 (not applicable), not specifying the degree of difficulty.

Altogether, 24 categories were selected: 10 from “Learning and applying knowledge”, three from “General tasks and demands”, five from “Communication”, four from “Self-care”, and two from “Major life areas”, as shown in Chart 1.

Collected data were tabulated in an Excel spreadsheet and compiled to characterize the children’s profiles and SLH manifestations and construct the category list. Data on the process of classifying the manifestations present in the reports were gathered for analysis.

Descriptive and bivariate analyses were performed. The descriptive analysis was based on the distribution of absolute and relative frequencies of categorical variables and the numerical synthesis of continuous variables. The ICF Activities and Participation categories were defined as the response variables, and the sex, age, educational attainment, and overall SLH diagnosis were defined as the explanatory variables. The qualifier .9 (not applicable) was removed for statistical analyses to make data synthesis easier.

The bivariate analysis used Fisher’s exact test for all categorical variables. This analysis required the recategorization and creation of new variables because of the few observations in certain categories of variables. “Educational attainment” was reclassified into two categories in the new variable: “Attends school” (yes for “Preschool education” and “Elementary school” and no for “Does not attend”). “Age” was symmetrically distributed (Kolmogorov-Smirnov test with p-value = 0.002) and was categorized according to the cutoff (median of the distribution), which was 37.5 months. Hence it was divided into two categories: the first one ≤ 37.5 months and the second one > 37.5 months. In all analyses, the level of significance was set at 5%.

## RESULTS

The descriptive analysis of sociodemographic data showed that 59.4% of participants were males, and 40.6% were females. Their mean age was 41.03 months, with a standard deviation of 19.77, a minimum of 14, and a maximum of 96. As for educational attainment, 19 participants (60.7%) attended preschool, five participants (14.3%) attended elementary school, and eight participants (25%) did not attend school. The analysis of the overall SLH diagnosis indicated a prevalence of language disorders associated with other conditions (60%), followed by developmental language disorder (40%).

The descriptive analysis of the preselected ICF Activities and Participation categories showed “Not reported” was a recurrent answer, with the highest percentages in Non-speech vocal expression (d331) performance (93.8%), Making decisions (d177) capacity (90.6%), Solving problems (d175) performance (65.6%) and capacity (87.5%), Communicating with - receiving - spoken messages (d310) performance (65.6%), and Eating (d550) capacity (65.6%). Twelve out of the 24 selected categories encompassed the Performance and Capacity qualifiers together.

The following “Learning and applying knowledge” categories had the highest percentage of descriptions as “some difficulty”: Acquiring language (d132) - performance (68.8%) and capacity (96.9%); Learning through actions with objects (d131) - capacity (75%); Acquiring concepts (d137) - capacity (62.5%); Focusing attention (d160) - capacity (59.4%).

The three selected “General tasks and demands” categories were described with a higher prevalence of “some difficulty”, as follows: Handling stress and other psychological demands (d240) - performance (59.4%); Undertaking a single task (d210) - performance (50%); Carrying out daily routine (d230) - performance (50%).

Of the five selected “Communication” categories, the following were described with a higher percentage of “some difficulty”, as follows: Speaking (d330) - performance (62.5%) and capacity (100%); Conversation (d350) - capacity (75%); Communicating with - receiving - spoken messages (d310) - capacity (53.1%). The following categories were described with a higher percentage of “no difficulties”: Producing nonverbal messages (d335) - capacity (59.4%) and Non-speech vocal expression (d331) - capacity (50%).

There were many “not reported” answers in “Self-care”. Washing oneself (d510), Caring for body parts (d520), and Dressing (d540) had only the performance qualifier, and they were all described as having “some difficulty” more often (34.4%). Eating (d550) was described as having “no difficulty” in 46.9% in performance and 34.4% in capacity.

In “Major life areas”, Preschool education (d815) and School education (d820) likewise only had performance. School education was described as “not applicable” in 87.5% of patients. Preschool education was described as “no difficulties” in 37.5% and as “some difficulties” in 31.3%.

The association analysis between ICF Activities and Participation categories and the patients’ sex had no statistically significant results.

The association analysis between ICF Activities and Participation categories and the patients’ ages, categorized based on the median, found a statistically significant result between age and Solving problems (d175) - performance, with a p-value of 0.045. Among participants with difficulties, 71.4% were ≤ 37.5 months old. The association analysis between ICF Activities and Participation categories and “School attendance” (yes and no) found a statistically significant result with Preschool education (d815) - performance, with a p-value of 0.003; all patients who had no difficulties attended school (Table 1).

**Table 1:** Association analysis between selected ICF variables and the participants’ sociodemographic data

**Chapter 1 - Learning and applying knowledge**												
	**Performance**			**Capacity**			**Performance**			**Capacity**		
	**Age (in months)**			**Age (in months)**			**Attends school**			**Attends school**		
**Watching (d110)**	≤37.5	>37.5	Total	≤37.5	>37.5	Total	Yes	No	Total	Yes	No	Total
	**N (%)**	**N (%)**		**N (%)**	**N (%)**		**N (%)**	**N (%)**		**N (%)**	**N (%)**	
No difficulties	9 (60.0)	6 (40.0)	15 (100.0)	-	-	-	8 (61.5)	5 (38.5)	13 (100.0)	-	-	-
Some difficulty	1 (25.0)	3 (75.0)	4 (100.0)	-	-	-	3 (100.0)	0 (0.0)	3 (100.0)	-	-	-
P-value	0.249			-			0.295			-		
**Listening (d115)**	≤37.5	>37.5	Total	≤37.5	>37.5	Total	Yes	No	Total	Yes	No	Total
	**N (%)**	**N (%)**		**N (%)**	**N (%)**		**N (%)**	**N (%)**		**N (%)**	**N (%)**	
No difficulties	9 (56.2)	7 (43.8)	16 (100.0)	8 (50.0)	8 (50.0)	16 (100.0)	8 (57.1)	6 (42.9)	15 (100.0)	10 (66.7)	5 (33.3)	15 (100.00)
Some difficulty	1 (100.0)	0 (0.0)	1 (100.0)	2 (40.0)	3 (60.0)	5 (100.0)	1 (100.0)	0 (0.0)	1 (100.0)	4 (80.0)	1 (20.0)	5 (100.0)
P-value	0.588			0.550			0.600			0.517		
**Copying (d130)**	≤37.5	>37.5	Total	≤37.5	>37.5	Total	Yes	No	Total	Yes	No	Total
	**N (%)**	**N (%)**		**N (%)**	**N (%)**		**N (%)**	**N (%)**		**N (%)**	**N (%)**	
No difficulties	-	-	-	5 (55.6)	4 (44.4)	9 (100.0)	-	-	-	8 (100.0)	0 (0.0)	8 (100.0)
Some difficulty	-	-	-	6 (75.0)	2 (25.0)	8 (100.0)	-	-	-	3 (50.0)	3 (50.0)	6 (100.0)
P-value	-			0.373			-			0.055		
**Learning through actions with objects (d131)**	≤37.5	>37.5	Total	≤37.5	>37.5	Total	Yes	No	Total	Yes	No	Total
	**N (%)**	**N (%)**		**N (%)**	**N (%)**		**N (%)**	**N (%)**		**N (%)**	**N (%)**	
No difficulties	-	-	-	4 (66.7)	2 (33.3)	6 (100.0)	-	-	-	3 (60.0)	2 (40.0)	5 (100.0)
Some difficulty	-	-	-	12 (50.0)	12 (50.0)	24 (100.0	-	-	-	16 (76.2)	5 (23.8)	23 (100.0)
P-value	-			0.395			-			0.411		
**Acquiring language (d132)**	≤37.5	>37.5	Total	≤37.5	>37.5	Total	Yes	No	Total	Yes	No	Total
	**N (%)**	**N (%)**		**N (%)**	**N (%)**		**N (%)**	**N (%)**		**N (%)**	**N (%)**	
No difficulties	1 (33.3)	2 (66.7)	3 (100.0)	0 (0.0)	1 (100.0)	1 (100.0)	2 (100.0)	0 (0.0)	2 (100.0)	0 (0.0)	1 (100.0)	1 (100.0)
Some difficulty	10 (45.5)	12 (54.5)	22 (100.0)	16 (51.6)	15 (48.4)	31 (100.0)	16 (76.2)	5 (23.8)	23 (100.0)	21(77.8)	6 (22.2)	27 (100.0)
P-value	0.593			0.500			0.605			0.250		
**Acquiring concepts (d137)**	≤37.5	>37.5	Total	≤37.5	>37.5	Total	Yes	No	Total	Yes	No	Total
	**N (%)**	**N (%)**		**N (%)**	**N (%)**		**N (%)**	**N (%)**		**N (%)**	**N (%)**	
No difficulties	-	-	-	0 (0.0)	4 (100.0)	4 (100.0)	-	-	-	4 (100.0)	0 (0.0)	4 (100.0)
Some difficulty	-	-	-	10 (50.0)	10 (50.0)	20 (100.0)	-	-	-	12 (66.7)	6(33.3)	18 (100.0)
P-value	-			0.094			-			0.249		
**Acquiring skills (d155)**	≤37.5	>37.5	Total	≤37.5	>37.5	Total	Yes	No	Total	Yes	No	Total
	**N (%)**	**N (%)**		**N (%)**	**N (%)**		**N (%)**	**N (%)**		**N (%)**	**N (%)**	
No difficulties	9 (69.2)	4 (30.8)	13 (100.0)	4 (57.1)	3 (42.9)	7 (100.0)	7 (58.3)	5 (41.7)	12 (100.0)	4 (66.7)	2 (33.3)	6 (100.0)
Some difficulty	4 (50.0)	4 (50.0)	8 (100.0)	2 (28.6)	5 (71.4)	7 (100.0)	6 (75.0)	2 (25.0)	8 (100.0)	6 (85.7)	1(14.3)	7 (100.0)
P-value	0.336			0.296			0.392			0.437		
**Focusing attention (d160)**	≤37.5	>37.5	Total	≤37.5	>37.5	Total	Yes	No	Total	Yes	No	Total
	**N (%)**	**N (%)**		**N (%)**	**N (%)**		**N (%)**	**N (%)**		**N (%)**	**N (%)**	
No difficulties	4 (66.7)	2 (33.3)	6 (100.0)	3 (60.0)	2 (40.0)	5 (100.0)	3 (60.0)	2 (40.0)	5 (100.0)	4 (100.0)	0 (0.0)	4 (100.0)
Some difficulty	4 (40.0)	6 (60.0)	10 (100.0)	10 (52.6)	9 (47.4)	19 (100.0)	8 (80.0)	2 (20.0)	10 (100.0)	12 (70.6)	5 (29.4)	17 (100.0)
P-value	0.304			0.585			0.407			0.304		
**Solving problems (d175)**	≤37.5	>37.5	Total	≤37.5	>37.5	Total	Yes	No	Total	Yes	No	Total
	**N (%)**	**N (%)**		**N (%)**	**N (%)**		**N (%)**	**N (%)**		**N (%)**	**N (%)**	
No difficulties	0 (0.0)	4 (100.0)	4 (100.0)	2 (66.7)	1 (33.3)	3 (100.0)	3 (75.0)	1 (25.0)	4 (100.0)	2 (66.7)	1 (33.3)	3 (100.0)
Some difficulty	5 (71.4)	2 (28.6)	7 (100.0)	1 (100.0)	0 (0.0)	1 (100.0)	3 (50.0)	3 (50.0)	6 (100.0)	1 (100.0)	0 (0.0)	1 (100.0)
P-value	**0.045**			0.750			0.452			0.750		
**Making decisions (d177)**	≤37.5	>37.5	Total	≤37.5	>37.5	Total	Yes	No	Total	Yes	No	Total
	**N (%)**	**N (%)**		**N (%)**	**N (%)**		**N (%)**	**N (%)**		**N (%)**	**N (%)**	
No difficulties	7 (63.6)	4 (36.4)	11 (100.0)	2 (66.7)	1 (33.3)	3 (100.0)	8 (80.0)	2 (20.0)	10 (100.0)	2 (66.7)	1 (33.3)	3 (100.0)
Some difficulty	2 (100.0)	0 (0.0)	2 (100.0)	-	-	-	0 (0.0)	2 (100.0)	2 (100.0)	-	-	-
P-value	0.462			-			0.091			-		
**Chapter 2 - General tasks and demands**												
	**Performance**			**Capacity**			**Performance**			**Capacity**		
	**Age (in months)**			**Age (in months)**			**Attends school**			**Attends school**		
**Undertaking a single task (d210)**	≤37.5	>37.5	Total	≤37.5	>37.5	Total	Yes	No	Total	Yes	No	Total
	**N (%)**	**N (%)**		**N (%)**	**N (%)**		**N (%)**	**N (%)**		**N (%)**	**N (%)**	
No difficulties	5 (33.3)	10 (66.7)	15 (100.0)	15 (55.6)	12 (44.4)	27 (100.0)	11 (84.8)	2 (15.4)	13 (100.0)	16 (69.6)	7 (30.4)	23 (100.0)
Some difficulty	10 (62.5)	6 (37.5)	16 (100.0)	1 (20.0)	4 (80.0)	5 (100.0)	10 (66.7)	5 (33.3)	15 (100.0)	5 (100.0)	0 (0.0)	5 (100.0)
P-value	0.103			0.166			0.258			0.207		
**Carrying out daily routine (d230)**	≤37.5	>37.5	Total	≤37.5	>37.5	Total	Yes	No	Total	Yes	No	Total
	**N (%)**	**N (%)**		**N (%)**	**N (%)**		**N (%)**	**N (%)**		**N (%)**	**N (%)**	
No difficulties	1 (25.0)	3 (75.0)	4 (100.0)	-	-	-	2 (66.7)	1 (33.3)	3 (100.0)	-	-	-
Some difficulty	10 (62.5)	6 (37.5)	16 (100.0)	-	-	-	11 (68.8)	5 (31.30	16 (100.0)	-	-	-
P-value	0.217			-			0.705			-		
**Handling stress and other psychological demands (d240)**	≤37.5	>37.5	Total	≤37.5	>37.5	Total	Yes	No	Total	Yes	No	Total
	**N (%)**	**N (%)**		**N (%)**	**N (%)**		**N (%)**	**N (%)**		**N (%)**	**N (%)**	
No difficulties	6 (46.2)	7 (53.8)	13 (100.0)	-	-	-	-	-	-	-	-	-
Some difficulty	-	-	-	-	-	-	9 (75.0)	3 (25.0)	12 (100.0)	-	-	-
P-value	-			-			-			-		
**Chapter 3 - Communication**												
	**Performance**			**Capacity**			**Performance**			**Capacity**		
	**Age (in months)**			**Age (in months)**			**Attends school**			**Attends school**		
**Communicating with - receiving - spoken messages (d310)**	≤37.5	>37.5	Total	≤37.5	>37.5	Total	Yes	No	Total	Yes	No	Total
	**N (%)**	**N (%)**		**N (%)**	**N (%)**		**N (%)**	**N (%)**		**N (%)**	**N (%)**	
No difficulties	5 (55.6)	4 (44.4)	9 (100.0)	5 (41.7)	7 (58.3)	12 (100.0)	6 (75.0)	2 (25.0)	8 (100.0)	8 (80.0)	2 (20.0)	10 (100.0)
Some difficulty	2 (100.0)	0 (0.0)	2 (100.0)	10 (58.8)	7 (41.2)	17 (100.0)	2 (100.0)	0 (0.0)	2 (100.0)	11 (73.3)	4 (26.7)	15 (100.0)
P-value	0.382			0.297			0.622			0.545		
**Speaking (d330)**	≤37.5	>37.5	Total	≤37.5	>37.5	Total	Yes	No	Total	Yes	No	Total
	**N (%)**	**N (%)**		**N (%)**	**N (%)**		**N (%)**	**N (%)**		**N (%)**	**N (%)**	
No difficulties	-	-	-	-	-	-	-	-	-	-	-	-
Some difficulty	11 (55.0)	9 (45.0)	20 (100.0)	16 (50.0)	16 (50.0)	32 (100.0)	14 (70.0)	6 (30.0)	20 (100.0)	21 (75.0)	7 (25.0)	28 (100.0)
P-value	-			-			-			-		
**Non-speech vocal expression (d331)**	≤37.5	>37.5	Total	≤37.5	>37.5	Total	Yes	No	Total	Yes	No	Total
	**N (%)**	**N (%)**		**N (%)**	**N (%)**		**N (%)**	**N (%)**		**N (%)**	**N (%)**	
No difficulties	2 (100.0)	-	2 (100.0)	7 (43.8)	9 (56.2)	16 (100.0)	1 (100.0)	0 (0.0)	1 (100.0)	11 (84.6)	2 (15.4)	13 (100.0)
Some difficulty	-	-	-	1 (100.0)	0 (0.0)	1 (100.0)	-	-	-	-	-	-
P-value	-			0.471			-			-		
**Producing nonverbal messages (d335)**	≤37.5	>37.5	Total	≤37.5	>37.5	Total	Yes	No	Total	Yes	No	Total
	**N (%)**	**N (%)**		**N (%)**	**N (%)**		**N (%)**	**N (%)**		**N (%)**	**N (%)**	
No difficulties	7 (70.0)	3 (30.0)	10 (100.0)	13 (68.4)	6 (31.6)	19 (100.0)	6 (66.7)	3 (33.3)	9 (100.0)	12 (75.0)	4 (25.0)	16 (100.0)
Some difficulty	1 (100.0)	0 (0.0)	1 (100.0)	0 (0.0)	1 (100.0)	1 (100.0)	1 (100.0)	0 (0.0)	1 (100.0)	1 (100.0)	0 (0.0)	1 (100.0)
P-value	0.727			0.350			0.700			0.765		
**Conversation (d350)**	≤37.5	>37.5	Total	≤37.5	>37.5	Total	Yes	No	Total	Yes	No	Total
	**N (%)**	**N (%)**		**N (%)**	**N (%)**		**N (%)**	**N (%)**		**N (%)**	**N (%)**	
No difficulties	-	-	-	3 (50.0)	3 (50.0)	6 (100.0)	-	-	-	3 (50.0)	3 (50.0)	6 (100.0)
Some difficulty	-	-	-	13 (54.2)	11 (45.8)	-	-	-	-	16 (80.0)	4 (20.0)	20 (100.0)
P-value	-			0.605			-			0.175		
**Chapter 5 - Self-care**												
	**Performance**			**Capacity**			**Performance**			**Capacity**		
	**Age (in months)**			**Age (in months)**			**Attends school**			**Attends school**		
**Washing oneself (d510)**	≤37.5	>37.5	Total	≤37.5	>37.5	Total	Yes	No	Total	Yes	No	Total
	**N (%)**	**N (%)**		**N (%)**	**N (%)**		**N (%)**	**N (%)**		**N (%)**	**N (%)**	
No difficulties	2 (66.7)	1 (33.3)	3 (100.0)	-	-	-	1 (50.0)	1 (50.0)	2 (100.0)	-	-	-
Some difficulty	7 (63.6)	4 (36.4)	11 (100.0)	-	-	-	7 (63.6)	4 (36.4)	11 (100.0)	-	-	-
P-value	0.725			-			0.641			-		
**Caring for body parts (d520)**	≤37.5	>37.5	Total	≤37.5	>37.5	Total	Yes	No	Total	Yes	No	Total
	**N (%)**	**N (%)**		**N (%)**	**N (%)**		**N (%)**	**N (%)**		**N (%)**	**N (%)**	
No difficulties	1 (33.3)	2 (66.7)	3 (100.0)	-	-	-	1 (50.0)	1 (50.0)	2 (100.0)	-	-	-
Some difficulty	7 (63.6)	4 (36.4)	11 (100.0)	-	-	-	8 (72.7)	3 (27.3)	11 (100.0)	-	-	-
P-value	0.385			-			0.538			-		
**Dressing (d540)**	≤37.5	>37.5	Total	≤37.5	>37.5	Total	Yes	No	Total	Yes	No	Total
	**N (%)**	**N (%)**		**N (%)**	**N (%)**		**N (%)**	**N (%)**		**N (%)**	**N (%)**	
No difficulties	0 (0.0)	2 (100.0)	2 (100.0)	-	-	-	1 (50.0)	1 (50.0)	2 (100.0)	-	-	-
Some difficulty	7 (63.6)	4 (36.4)	11 (100.0)	-	-	-	7 (63.6)	4 (36.4)	11 (100.0)	-	-	-
P-value	0.192			-			0.641			-		
**Eating (d550)**	≤37.5	>37.5	Total	≤37.5	>37.5	Total	Yes	No	Total	Yes	No	Total
	**N (%)**	**N (%)**		**N (%)**	**N (%)**		**N (%)**	**N (%)**		**N (%)**	**N (%)**	
No difficulties	10 (66.7)	5 (33.3)	15 (100.0)	8 (72.7)	3 (27.3)	11 (100.0)	8 (53.3)	7 (46.7)	15 (100.0)	5 (50.0)	5 (50.0)	10 (100.0)
Some difficulty	2 (50.0)	2 (50.0)	4 (100.0)	-	-	-	3 (100.0)	0 (0.0)	3 (100.0)	-	-	-
P-value	0.475			-			0.202			-		
**Chapter 8 - Major life areas**												
	**Performance**			**Capacity**			**Performance**			**Capacity**		
	**Age (in months)**			**Age (in months)**			**Attends school**			**Attends school**		
**Preschool education (d815)**	≤37.5	>37.5	Total	≤37.5	>37.5	Total	Yes	No	Total	Yes	No	Total
	**N (%)**	**N (%)**		**N (%)**	**N (%)**		**N (%)**	**N (%)**		**N (%)**	**N (%)**	
No difficulties	5 (41.7)	7 (58.3)	12 (100.0)	-	-	-	12 (100.0)	0 (0.0)	12 (100.0)	-	-	-
Some difficulty	7 (70.0)	3 (30.0)	10 (100.0)	-	-	-	4 (40.0)	6 (60.0)	10 (100.0	-	-	-
P-value	0.185			-			**0.003**			-		
**School education (d820)**	≤37.5	>37.5	Total	≤37.5	>37.5	Total	Yes	No	Total	Yes	No	Total
	**N (%)**	**N (%)**		**N (%)**	**N (%)**		**N (%)**	**N (%)**		**N (%)**	**N (%)**	
No difficulties	-	1 (100.0)	1 (100.0)	-	-	-	1 (100.0)	-	1 (100.0)	-	-	-
Some difficulty	-	3 (100.0)	3 (100.0)	-	-	-	3 (100.0)	-	3 (100.0)	-	-	-
P-value	-			-			-			-		

N = number of participants, data varies according to information characteristics and missing data; Fisher’s exact test

The association between ICF categories and “SLH diagnosis” (developmental language disorder ad language disorders associated with other conditions) found statistical significance between SLH diagnosis and Conversation (d350) - capacity (p = 0.013); 77.3% of participants with difficulties had an SLH diagnosis of language disorder associated with other conditions (Table 2).

**Table 2:** Association analysis between selected ICF variables and speech-language-hearing diagnosis

**Chapter 1 - Learning and applying knowledge**							
	**Performance**			**Capacity**			
	SLH diagnosis			SLH diagnosis			
**Watching (d110)**	DLD**	LD***	Total	DLD**	LD***	Total	
	**N (%)**	**N (%)**		**N (%)**	**N (%)**		
No difficulties	6 (42.9)	8 (57.1)	14.0	-	-	-	
Some difficulty	0 (0.0)	4 (100.0)	4 (100.0)	-	-	-	
P-value	0.162			-			
**Listening (d115)**	DLD**	LD***	Total	DLD**	LD***	Total	
	**N (%)**	**N (%)**		**N (%)**	**N (%)**		
No difficulties	3 (20.0)	12 (80.0)	15 (100.0)	7 (46.7)	8 (53.3)	15 (100.0)	
Some difficulty	1 (100.0)	0 (0.0)	1 (100.0)	2 (40.0)	3 (60.0)	5 (100.0)	
P-value	0.250			0.604			
**Copying (d130)**	DLD**	LD***	Total	DLD**	LD***	Total	
	**N (%)**	**N (%)**		**N (%)**	**N (%)**		
No difficulties	-	-	-	5 (55.6)	4 (44.4)	9 (100.0)	
Some difficulty	-	-	-	1 (14.3)	6 (85.7)	7 (100.0)	
P-value	-			0.121			
**Learning through actions with objects (d131)**	DLD**	LD***	Total	DLD**	LD***	Total	
	**N (%)**	**N (%)**		**N (%)**	**N (%)**		
No difficulties	-	-	-	2 (33.3)	4 (66.7)	6 (100.0)	
Some difficulty	-	-	-	9 (40.9)	13 (59.1)	22 (100.0)	
P-value	-			0.561			
**Acquiring language (d132)**	DLD**	LD***	Total	DLD**	LD***	Total	
	**N (%)**	**N (%)**		**N (%)**	**N (%)**		
No difficulties	3 (100.0)	0 (0.0)	3 (100.0)	1 (100.0)	0 (0.0)	1 (100.0)	
Some difficulty	7 (35.0)	13 (65.0)	20 (100.0)	11 (37.9)	18 (62.1)	29 (100.0)	
P-value	0.068			0.400			
**Acquiring concepts (d137)**	DLD**	LD***	Total	DLD**	LD***	Total	
	**N (%)**	**N (%)**		**N (%)**	**N (%)**		
No difficulties	-	-	-	2 (50.0)	2 (50.0)	4 (100.0)	
Some difficulty	-	-	-	7 (36.8)	12 (63.2)	19 (100.0)	
P-value	-			0.517			
**Acquiring skills (d155)**	DLD**	LD***	Total	DLD**	LD***	Total	
	**N (%)**	**N (%)**		**N (%)**	**N (%)**		
No difficulties	7 (58.3)	5 (41.7)	12 (100.0)	3 (42.9)	4 (57.1)	7 (100.0)	
Some difficulty	3 (37.5)	5 (62.5)	8 (100.0)	4 (57.1)	3 (42.9)	7 (100.0)	
P-value	0.325			0.500			
**Focusing attention (d160)**	DLD**	LD***	Total	DLD**	LD***	Total	
	**N (%)**	**N (%)**		**N (%)**	**N (%)**		
No difficulties	3 (60.0)	2 (40.0)	5 (100.0)	3 (60.0)	2 (40.0)	5 (100.0)	
Some difficulty	5 (50.0)	5 (50.0)	10 (100.0)	5 (27.8)	13 (72.2)	18 (100.0)	
P-value	0.573			0.208			
**Solving problems (d175)**	DLD**	LD***	Total	DLD**	LD***	Total	
	**N (%)**	**N (%)**		**N (%)**	**N (%)**		
No difficulties	2 (50.0)	2 (50.0)	4 (100.0)	0 (0.0)	3 (100.0)	3 (100.0)	
Some difficulty	2 (33.3)	4 (66.7)	6 (100.0)	1 (100.0)	0 (0.0)	1 (100.0)	
P-value	0.548			0.250			
**Making decisions (d177)**	DLD**	LD***	Total	DLD**	LD***	Total	
	**N (%)**	**N (%)**		**N (%)**	**N (%)**		
No difficulties	6 (54.5)	5 (45.5)	11 (100.0)	1 (33.3)	2 (66.7)	3 (100.0)	
Some difficulty	0 (0.0)	1 (100.0)	1 (100.0)	-	-	-	
P-value	0.500			-			
**Chapter 2 - general tasks and demands**							
	**Performance**			**Capacity**			
	SLH diagnosis			SLH diagnosis			
**Undertaking a single task (d210)**	DLD**	LD***	Total	DLD**	LD***	Total	
	**N (%)**	**N (%)**		**N (%)**	**N (%)**		
No difficulties	6 (42.9)	8 (57.1)	14 (100.0)	9 (36.0)	16 (64.0)	25 (100.0)	
Some difficulty	6 (40.0)	9 (60.0)	15 (100.0)	3 (60.0)	2 (40.0)	5 (100.0)	
P-value	0.587			0.304			
**Carrying out daily routine (d230)**	DLD**	LD***	Total	DLD**	LD***	Total	
	**N (%)**	**N (%)**		**N (%)**	**N (%)**		
No difficulties	2 (50.0)	2 (50.0)	4 (100.0)	-	-	-	
Some difficulty	7 (46.7)	8 (53.3)	15 (100.0)	-	-	-	
P-value	0.667			-			
**Handling stress and other psychological demands (d240)**	DLD**	LD***	Total	DLD**	LD***	Total	
	**N (%)**	**N (%)**		**N (%)**	**N (%)**		
No difficulties	-	-	-	-	-	-	
Some difficulty	6 (50.0)	6 (50.0)	12 (100.0)	-	-	-	
P-value	-			-			
**Chapter 3 - Communication**							
	**Performance**			**Capacity**			
	SLH diagnosis			SLH diagnosis			
**Communicating with - receiving - spoken messages (d310)**	DLD**	LD***	Total	DLD**	LD***	Total	
	**N (%)**	**N (%)**		**N (%)**	**N (%)**		
No difficulties	4 (50.0)	4 (50.0)	8 (100.0)	4 (36.4)	7 (63.6)	11 (100.0)	
Some difficulty	1 (50.0)	1 (50.0)	2 (100.0)	7 (43.8)	9 (56.3)	16 (100.0)	
P-value	0.778			0.508			
**Speaking (d330)**	DLD**	LD***	Total	DLD**	LD***	Total	
	**N (%)**	**N (%)**		**N (%)**	**N (%)**		
No difficulties	-	-	-	-	-	-	
Some difficulty	8 (44.4)	10 (55.6)	18 (100.0)	12 (40.0)	18 (60.0)	30 (100.0)	
P-value	-			-			
**Non-speech vocal expression (d331)**	DLD**	LD***	Total	DLD**	LD***	Total	
	**N (%)**	**N (%)**		**N (%)**	**N (%)**		
No difficulties	0 (0.0)	2 (100.0)	2 (100.0)	3 (20.0)	12 (80.0)	15 (100.0)	
Some difficulty	-	-	-	0 (0.0)	1 (100.0)	1 (100.0)	
P-value	-			0.813			
**Producing nonverbal messages (d335)**	DLD**	LD***	Total	DLD**	LD***	Total	
	**N (%)**	**N (%)**		**N (%)**	**N (%)**		
No difficulties	3 (30.0)	7 (70.0)	10 (100.0)	7 (38.9)	11 (61.1)	18 (100.0)	
Some difficulty	0 (0.0)	1 (100.0)	1 (100.0)	0 (0.0)	1 (100.0)	1 (100.0)	
P-value	0.727			0.632			
**Conversation (d350)**	DLD**	LD***	Total	DLD**	LD***	Total	
	**N (%)**	**N (%)**		**N (%)**	**N (%)**		
No difficulties	-	-	-	5 (83.3)	1 (16.7)	6 (100.0)	
Some difficulty	-	-	-	5 (22.7)	17 (77.3)	22 (100.0)	
P-value	-			**0.013**			
**Chapter 5 - Self-care**							
	**Performance**			**Capacity**			
	SLH diagnosis			SLH diagnosis			
**Washing oneself (d510)**	DLD**	LD***	Total	DLD**	LD***	Total	
	**N (%)**	**N (%)**		**N (%)**	**N (%)**		
No difficulties	3 (100.0)	0 (0.0)	3 (100.0)	-	-	-	
Some difficulty	5 (50.0)	5 (50.0)	10 (100.0)	-	-	-	
P-value	0.196			-			
**Caring for body parts (d520)**	DLD**	LD***	Total	DLD**	LD***	Total	
	**N (%)**	**N (%)**		**N (%)**	**N (%)**		
No difficulties	3 (100.0)	0 (0.0)	3 (100.0)	-	-	-	
Some difficulty	6 (54.5)	5 (45.5)	11 (100.0)	-	-	-	
P-value	0.231			-			
**Dressing (d540)**	DLD**	LD***	Total	DLD**	LD***	Total	
	**N (%)**	**N (%)**		**N (%)**	**N (%)**		
No difficulties	2 (100.0)	0 (0.0)	2 (100.0)	-	-	-	
Some difficulty	5 (50.0)	5 (50.0)	10 (100.0)	-	-	-	
P-value	0.318			-			
**Eating (d550)**	DLD**	LD***	Total	DLD**	LD***	Total	
	**N (%)**	**N (%)**		**N (%)**	**N (%)**		
No difficulties	8 (57.1)	6 (42.9)	14 (100.0)	5 (50.0)	5 (50.0)	10 (100.0)	
Some difficulty	0 (0.0)	4 (100.0)	4 (100.0)	-	-	-	
P-value	0.069			-			
**Chapter 8 - Major life areas**							
	**Performance**			**Capacity**			
	SLH diagnosis			SLH diagnosis			
**Preschool education (d815)**	DLD**	LD***	Total	DLD**	LD***	Total	
	**N (%)**	**N (%)**		**N (%)**	**N (%)**		
No difficulties	5 (45.5)	6 (54.5)	11 (100.0)	-	-	-	
Some difficulty	3 (30.0)	7 (70.0)	10 (100.0)	-	-	-	
P-value	0.392			-			
**School education (d820)**	DLD**	LD***	Total	DLD**	LD***	Total	
	**N (%)**	**N (%)**		**N (%)**	**N (%)**		
No difficulties	0 (0.0)	1 (100.0)	1 (100.0)	-	-	-	
Some difficulty	3 (100.0)	0 (0.0)	3 (100.0)	-	-	-	
P-value	0.250			-			

N=number of participants, data varies according to information characteristics and missing data;

** Developmental language disorder;

*** Language disorder associated with other conditions; Fisher’s exact test

**Caption:** SLH = Speech-language-hearing

## DISCUSSION

The diversity of ICF Activities and Participation categories found in this study led to reflections on the impacts oral language disorders may have on child development, especially regarding activity limitations and participation restrictions. Hence, the study aimed to identify ICF Activities and Participation categories and their associations with age, sex, educational attainment, and overall SLH diagnosis in the context of a pediatric language outpatient center.

As for sociodemographic data, the study shows that most participants were males -similar results to that of other studies that associated the prevalence of child language changes with males due to neurological, hormonal, and social factors^(14-19)^. Concerning the findings related to age and educational attainment in the sample, a study on the prevalence of SLH changes in children verified a prevalence of communication changes in preschoolers (48.7% of participants aged 36 to 72 months)^(16)^. It must be pointed out that the present study has a wide age range, from 14 to 96 months - a much wider range to consider and refer to developmental stages and communication changes.

The predominating SLH diagnosis of language disorder associated with other conditions corroborates a study on diagnostic profiles at an SLH outpatient center in the field of child language. It verified a high prevalence of comorbidities (76.9%), with the greatest occurrence of associations with neurological diseases and neuropsychomotor developmental delay^(17)^.

Regarding the ICF Activities and Participation categories, the results show that preselecting chapters and categories related to pediatric oral language changes favors their application, as all selected chapters and categories were included in this study. The importance of considering factors associated with activity limitations and participation restrictions is reflected in the multiple categories also found in other studies^(20-23)^ - which demonstrates the diversity of SLH manifestations present in a language diagnosis. A study on expectations and results found after speech and language therapy in preschoolers verified that most concerns, expectations, and progress perceived by the parents and physicians were related to activity limitations^(21)^. This shows the importance of integrating aspects of functioning (particularly those related to activity limitations and participation restrictions) in SLH practice regarding child oral language disorders.

A study on the characterization of outpatients’ performance in SLH aspects according to the ICF version for children and youth (ICF-CY) verified the prevalence of difficulties described in categories related to language, learning, and school issues^(20)^. The study reported a greater occurrence of difficulties described in categories of chapters on basic learning and knowledge application, namely: Learning not read (d140) - performance; Focusing attention (d161) - performance; Writing (d170) - capacity; and Learning to write (d145) - capacity. The studies coincide with the prevalence of difficulties described in “Learning and applying knowledge”. However, they differ regarding the categories described with the highest percentage of “some difficulty”. Such a difference can be explained by the setting, characterization of the sample, age range, and changes patients had in each study. The cited study analyzed medical records of patients treated at an assessment outpatient center, aged 5 to 16 years, whose changes may not have been related to oral languages; moreover, the patients could have other types of changes, such as in reading and writing. As for the characterization of the sample, the exclusion criteria in that study encompassed patients with suspected or confirmed diagnoses of global developmental delay.

On the other hand, a study identified ICF categories in cases of language and speech disorders and found that the most frequent Activities and Participation categories - Communicating with - receiving - spoken messages (d310), Speaking (d330), Acquiring skills (d155), and Conversation (d350) - were related to the chapters on Basic learning and Communication^(22)^. This is similar to the results in the present study regarding the chapters with the most recurrent description of difficulties, as well as Speaking (d330) and Conversation (d350), which were also described with a higher percentage of “some difficulties”.

Studies associating categories relevant to oral language changes with personal factors and clinical diagnoses help understand health conditions. The analyses in this study verified an association between age and Solving problems (d175) - performance, in which most participants with difficulties were ≤ 37.5 months old. This can be explained by the development of autonomy, social skills, entering school, or a combination of two or more factors. Thus, regardless of the language diagnosis, developmental factors play a major role. Another important factor is the school experience, which is essential to social and educational acquisition in the first years at school^(24)^. Educational settings favor the progress of cognitive stages, strengthening their abilities to deal with adversities and encouraging creative solutions to their problems^(24)^.

The association between attending school and Preschool education (d815) - performance (in which all participants who did not have difficulties attended school) can be explained by the fact that the complaints of preschoolers’ parents were not yet related to school issues - which begin in school age. After they enter school, parents tend to broaden their perception of complaints related to school difficulties. A study in schoolchildren’s relatives found that the main complaints that motivated referrals to the clinic were related to writing and reading difficulties, while complaints on psychological/behavioral aspects were less frequent^(25)^.

Another study, conducted in an SLH assessment outpatient center, verified an association between educational attainment and the ICF Activities and Participation factor named “Family/school”, which comprises “Household relationships - performance” and “School education - performance”. This factor obtained higher scores among older patients who attended elementary or middle school, which are explained by the greater school demands on elementary school or more advanced students^(26)^. Even though the studies are different regarding analysis variables, school age can be related to the greatest school demands and difficulties observed in the cited study. This reflects the importance that SLH practices encompass oral and written language, even before starting formal education, because mastering the oral linguistic system and developing metacognitive and metalinguistic skills are essential to learning to read and write^(27)^.

The association verified between SLH diagnoses and Conversation d350 - capacity (in which most participants with difficulties were diagnosed with language disorders associated with other conditions) can be explained by the patients’ profile in the service. The hospital to which the outpatient center in question belongs provides medium- and high-complexity care and is a reference in referrals to services such as neurology and genetics and receives mostly patients with autism spectrum disorder and Down syndrome. Pragmatic skill impairments are frequent in these conditions, which causes interaction, social communication, and conversation difficulties^(28)^.

Because the analysis was based on secondary data collected from medical records, the limitation of this study involves missing information, as demonstrated in the many “not reported” answers in the descriptive analysis of ICF categories. This can be explained by medical history survey and assessment tools used in the service, which may have had little association with ICF functioning aspects. This indicates the need for developing standardized clinical instruments with categories relevant to child language changes, enabling their implementation in clinical practice at the service and minimizing the loss of information in medical record analysis. The study also has limitations regarding its sample size. However, it involved an outpatient center whose population is not large, and the sample was representative of the service’s profile.

Concerning advancements, the study made it possible to identify the main categories relevant to pediatric oral language changes, understand activity limitations and participation restrictions resulting from this condition, and implement the list of categories constructed at the service. Hence, it enabled the application of ICF in its clinical practice and the development of a future longitudinal study with the same population, expanding the use of qualifiers that specify the degree of difficulty to compare before and after SLH therapy. This study can also motivate SLH therapists of other services whose patients have a similar profile to include and operationalize ICF in the clinical assistance flow.

The study results show the importance of using ICF as a tool to integrate the impacts of language changes on functioning and the expansion of the biopsychosocial approach in SLH clinical practice.

## CONCLUSION

The study selected 24 ICF Activities and Participation categories, 12 of which encompassed both performance and capacity qualifiers in children with oral language disorders undergoing SLH therapy at a public outpatient service. Difficulties were described most often in the categories of Learning and applying knowledge and Communication and were related to activities such as Acquiring language, Learning through actions with objects, Speaking, and Conversation.

It also found statistically significant associations between sociodemographic data and SLH diagnoses and ICF Activities and Participation categories.
