# Supplementary material for: Activities and participation of children with language disorders in outpatient care according to the ICF
Source: Codas. 2023 Aug 4;35(4):e20220007. doi: 10.1590/2317-1782/20232022007en (PMC10446747; doi:10.1590/2317-1782/20232022007en)
Supplement: Translation

# Translation: Atividades e participação de crianças com transtornos de linguagem em atendimento ambulatorial segundo a CIF

**DOI:** 10.1590/2317-1782/20232022007pt

**Authors:** Gabriela Damaris Ribeiro Nogueira, Stela Maris Aguiar Lemos, Denise Brandão de Oliveira e Britto

**Affiliations:** 1 Departamento de Fonoaudiologia, Faculdade de Medicina, Universidade Federal de Minas Gerais - UFMG - Belo Horizonte (MG), Brasil.

**Keywords:** Linguagem Infantil, Desenvolvimento da Linguagem, Transtornos do Desenvolvimento da Linguagem, Criança, Classificação Internacional de Funcionalidade, Incapacidade e Saúde

## Abstract

**Objetivo:**

Identificar as principais categorias do componente Atividades e Participação da Classificação Internacional de Funcionalidade, Incapacidade e Saúde, bem como os possíveis qualificadores de Capacidade e Desempenho, e verificar suas associações com idade, sexo, escolaridade e diagnóstico fonoaudiológico em crianças atendidas em um ambulatório de linguagem oral.

**Método:**

Trata-se de estudo observacional analítico e transversal, realizado com dados secundários de 32 prontuários de crianças com maioria do sexo masculino, média de idade de 41,03 meses, em ensino infantil e transtorno de linguagem associado a outras condições. Foi realizada a codificação das principais manifestações fonoaudiológicas segundo as categorias do componente Atividades e Participação pré-selecionadas e realizadas análises estatísticas descritivas e bivariadas e uso do teste Exato de Fisher com nível de significância de 5%.

**Resultados:**

A análise descritiva das categorias pré-selecionadas permitiu verificar um número alto de respostas “Não informado”, com maior porcentagem em expressão vocal sem fala (d331) desempenho (93,8%), tomar decisões (d177) capacidade (90,6%), resolver problemas (d175) desempenho (65,6%) e capacidade (87,5%), recepção de mensagens orais (d310) desempenho (65,6%) e comer (d550) capacidade (65,6%). Dentre as 24 categorias selecionadas, 12 contemplaram conjuntamente os qualificadores de Desempenho e Capacidade. Houve associação com significância estatística entre as categorias (d175, d815 e d350) com dados sociodemográficos e diagnósticos fonoaudiológicos.

**Conclusão:**

Foram identificadas dificuldades em categorias do componente Atividades e Participação e associações com significância estatística entre elas e os dados sociodemográficos e diagnósticos fonoaudiológicos, evidenciando impactos dos transtornos de linguagem oral nas atividades e participação de crianças atendidas em um serviço ambulatorial.

## INTRODUÇÃO

A linguagem é um sistema de comunicação fundamental para a funcionalidade humana e por meio dela o ser humano interage na sociedade. A aquisição e o refinamento das habilidades de fala e linguagem no decorrer do desenvolvimento infantil, permitem que a criança exerça maior controle sobre sua vida por meio da expressão de suas necessidades, sentimentos e ideias. Comprometimentos na linguagem infantil podem gerar impacto ou prejuízo ao desenvolvimento psicossocial e cognitivo, bem como à qualidade de vida da criança^(1-3)^.

A avaliação da linguagem infantil e o planejamento das estratégias terapêuticas na reabilitação não devem se restringir aos aspectos orgânicos, mas integrar a influência dos aspectos ambientais, psicossociais e cognitivos. Tal abordagem permite uma compreensão mais ampla da linguagem, que considera seu uso nos diferentes contextos da vida e a maneira que esta molda a funcionalidade da criança^(4)^.

A Classificação Internacional de Funcionalidade, Incapacidade e Saúde (CIF) é uma classificação pertencente à Família de Classificações Internacionais da Organização Mundial de Saúde (OMS), que se propõe a descrever a saúde e seus estados relacionados e proporcionar uma linguagem unificada e padronizada^(5)^. Dessa forma, a ferramenta permite classificar, por meio de uma perspectiva biopsicossocial, a funcionalidade, a incapacidade e os fatores contextuais associados às condições de saúde^(5)^.

O uso da CIF como ferramenta para monitorar a evolução da funcionalidade contribui para identificar, compreender e monitorar os impactos que o comprometimento de linguagem pode representar ao desenvolvimento global e social da criança, além de favorecer uma assistência fundamentada na atenção e cuidado integral à saúde e seus estados relacionados. Ao auxiliar na identificação dos aspectos da funcionalidade, principalmente aqueles relacionados às limitações de atividades e restrições de participação, a caracterização realizada por meio da CIF pode auxiliar no planejamento de intervenções para a superação das barreiras nos diferentes contextos da vida^(2)^.

A American Speech-Language-Hearing Association (ASHA) recomenda a incorporação da CIF em todos os componentes de atenção à saúde a fim de integrar, nas decisões clínicas, a influência dos fatores contextuais à funcionalidade e promover o desenvolvimento de objetivos funcionais e práticas colaborativas^(6)^.

Diante de sua complexidade e extensão, assim como da carência de estudos e ferramentas de referência, evidencia-se a necessidade de ampliar o uso da CIF na prática fonoaudiológica, por meio do desenvolvimento de instrumentos clínicos padronizados que norteiem e auxiliem sua aplicação clínica^(7)^. Além disso, o desenvolvimento de estudos que associem as categorias relevantes às alterações de linguagem oral, com os fatores pessoais e diagnósticos clínicos, favorece uma compreensão mais ampla do comprometimento de linguagem, e considera suas repercussões individuais e sociais.

A CIF é estruturada em duas partes. A primeira, Funcionalidade e Incapacidade, inclui os componentes Funções e Estruturas do Corpo e, Atividades e Participação. A segunda parte traz as informações do contexto, incluindo os componentes Fatores Ambientais e Fatores Pessoais. Os aspectos positivos (integridade, funcional e estrutural, atividades e participação) são denominados Funcionalidade. Os aspectos negativos (deficiência, limitação da atividade ou restrição da participação) constituem a Incapacidade. Em relação aos fatores ambientais, os aspectos positivos são os facilitadores e os aspectos negativos são barreiras^(5)^.

No presente estudo, o foco será o componente Atividades e Participação, que contempla os qualificadores de Capacidade e Desempenho e inclui domínios que indicam os aspectos da funcionalidade no nível individual e social, e, por isso, representa a “matriz da informação” gerada pela CIF^(5)^. Por entender que as alterações de linguagem em crianças podem comprometer execuções de tarefas ou ações (Atividades) e o envolvimento em situações de vida diária (Participação) configurando limitações e restrições, respectivamente, é de suma importância investigar as interações entre os construtos Linguagem e Funcionalidade.

Portanto, os objetivos do presente estudo foram identificar as principais categorias, referentes ao componente Atividades e Participação da CIF, bem como os possíveis qualificadores de Capacidade e Desempenho, e verificar suas associações com idade, sexo, escolaridade e diagnóstico fonoaudiológico global em crianças atendidas em um ambulatório de linguagem oral.

## MÉTODO

Esta pesquisa foi aprovada pelo Comitê de Ética em Pesquisa da Universidade Federal de Minas Gerais sob número 3.172.707 e CAAE: 02470618.1.0000.5149.

Trata-se de estudo observacional analítico, de caráter transversal, com base na análise de dados secundários, coletados em prontuários de pacientes atendidos em um serviço fonoaudiológico ambulatorial pertencente a um hospital da rede pública.

O serviço em questão é um ambulatório de terapia de linguagem oral infantil, que presta assistência a crianças com Transtornos de Linguagem associados à Síndrome de Down e outras condições genéticas ou sensoriais; Transtorno do Espectro Autista; Transtorno do Desenvolvimento da Linguagem e Transtornos dos Sons da Fala.

A amostra refere-se aos dados coletados de 32 prontuários de pacientes, configurando a totalidade dos atendidos no período de agosto de 2018 a março de 2021, com faixa etária entre 14 meses e 8 anos e que apresentavam diagnóstico de transtorno de linguagem oral infantil. Todos os responsáveis pelos participantes da pesquisa assinaram o Termo de Consentimento Livre e Esclarecido, e autorizaram o acesso aos prontuários dos participantes.

Inicialmente, foram coletados os seguintes dados nos relatórios de avaliação inicial: sexo, data de nascimento, escolaridade e diagnóstico fonoaudiológico global. Em seguida, foi realizada a classificação das principais manifestações e diagnósticos fonoaudiológicos segundo o componente Atividades e Participação da CIF, utilizando seus qualificadores de Capacidade e Desempenho.

Foram consideradas as manifestações e hipóteses diagnósticas fonoaudiológicas relacionadas às alterações de linguagem, em seus aspectos receptivos, expressivos e seus subsistemas fonológico, morfossintático, pragmático e semântico, assim como dos aspectos do desenvolvimento cognitivo, descritos nos relatórios conforme os resultados dos seguintes testes: Protocolo de Observação Comportamental (PROC)^(8)^, Avaliação do Desenvolvimento da Linguagem (ADL)^(9)^, Protocolo de Observação de Comportamentos de crianças até 6 anos de idade ^(10)^, Teste de Linguagem Infantil ABFW^(11)^, Teste de Vocabulário por Figuras USP (TVfusp)^(12)^, Teste de Vocabulário Auditivo (TVAud) e Teste de Vocabulário Expressivo (TVExp)^(13)^. Foram considerados também os resultados da avaliação auditiva, quando presentes.

Para a definição das categorias da CIF, foi realizada a pré-seleção dos capítulos do componente Atividades e Participação que se relacionam às alterações de linguagem oral infantil. Vale destacar que a pré-seleção foi feita de forma aberta, ou seja, considerando a descrição das manifestações e diagnósticos fonoaudiológicos elencados nos relatórios. Foram incluídos cinco no presente estudo, sendo eles: “Aprendizagem e aplicação de conhecimento”, “Tarefas e demandas gerais”, “Comunicação”, “Cuidado pessoal” e “Áreas principais da vida” (Quadro 1).

**Quadro 1:** Categorias selecionadas do componente Atividades e Participação da CIF

Capítulos	Categorias
Capítulo 1. Aprendizagem e aplicação de conhecimento	d110 - Observar
	d115 - Ouvir
	d130 - Imitar
	d131 - Aprender por meio de ações com objetos
	d132 - Aquisição de linguagem
	d137 - Aquisição de conceitos
	d155 - Aquisição de habilidades
	d160 - Concentrar a atenção
	d175 - Resolver problemas
	d177 - Tomar decisões
Capítulo 2. Tarefas e demandas gerais	d230 - Realizar a rotina diária
	d240 - Lidar com o estresse e outras demandas psicológicas
Capítulo 3. Comunicação	d310 - Comunicação-recepção de mensagens orais
	d330 - Fala
	d331 - Expressão vocal sem fala
	d335 - Produção de mensagens não verbais
	d350 - Conversação
Capítulo 5. Cuidado pessoal	d510 - Lavar-se
	d520 - Cuidado das partes do corpo
	d540 - Vestir-se
	d550 - Comer
Capítulo 8. Áreas principais da vida	d815 - Educação infantil
	d820 - Educação escolar

Após a definição dos capítulos, foi realizada a seleção do conjunto de categorias de cada um destes e posterior codificação dos achados nos relatórios, com os respectivos qualificadores de Desempenho e Capacidade. Para o presente estudo foram utilizados apenas os qualificadores .0 (nenhuma dificuldade), .8 (dificuldade não especificada) e .9 (não aplicável), não especificando o grau de dificuldade.

Foram selecionadas 24 categorias: dez do capítulo “Aprendizagem e aplicação de conhecimento”, três do capítulo “Tarefas e demandas gerais”, cinco do capítulo “Comunicação”, quatro do capítulo “Cuidado pessoal” e duas do capítulo “Áreas principais da vida”, conforme descrito no Quadro 1.

Os dados coletados foram tabulados em planilha construída no programa *Excel* e compilados para caracterização do perfil, das manifestações fonoaudiológicas e construção da lista de categorias. Os dados referentes ao processo de classificação das manifestações presentes nos relatórios foram compilados para análise.

Foram realizadas análises descritiva e bivariada. A análise descritiva foi realizada por meio de distribuição de frequência absoluta e relativa das variáveis categóricas, e de síntese numérica das variáveis contínuas. Foram definidas como variáveis resposta as categorias do componente Atividades e Participação da CIF e como as variáveis explicativas sexo, idade, escolaridade e diagnóstico fonoaudiológico global. Vale destacar que para as análises estatísticas o qualificador .9 (não aplicável) foi retirado para facilitar a síntese dos dados.

Na análise bivariada foi utilizado o teste Exato de Fisher para todas as variáveis categóricas. Para a realização da análise bivariada foi necessário realizar a recategorização e criação de novas variáveis, devido ao pequeno número de observações em determinadas categorias das variáveis. A variável “Escolaridade” foi reclassificada em duas categorias na nova variável: “Frequenta escola” (sim para “Ensino infantil” e “Ensino fundamental” e não para “Não frequenta”). A variável “Idade” teve distribuição assimétrica (Teste de Kolmogorov-Smirnov com valor-p=0,002) e optou-se por categorizá-la usando como ponto de corte a mediana da distribuição, que foi de 37,5 meses, sendo esta realizada em duas categorias, sendo a primeira ≤37,5 meses e a segunda categoria >37,5 meses. Para todas as análises foi considerado o nível de significância de 5%.

## RESULTADOS

A análise descritiva dos dados sociodemográficos revelou que 59,4% dos participantes pertenciam ao sexo masculino e 40,6% ao sexo feminino. A média de idade foi de 41,03 meses, desvio padrão 19,77, mínimo 14 e máximo 96. Em relação à escolaridade, 19 participantes (60,7%) cursavam o ensino infantil, cinco participantes (14,3%) cursavam o ensino fundamental e oito participantes (25%) não frequentavam a escola. A análise do diagnóstico fonoaudiológico global permitiu verificar prevalência de transtorno de linguagem associado a outras condições (60%), seguido de transtorno do desenvolvimento da linguagem (40%).

Quanto ao componente Atividades e Participação da CIF, a análise descritiva das categorias pré-selecionadas permitiu verificar um número alto de respostas “Não informado”, com maior porcentagem em expressão vocal sem fala (d331) desempenho (93,8%), tomar decisões (d177) capacidade (90,6%), resolver problemas (d175) desempenho (65,6%) e capacidade (87,5%), recepção de mensagens orais (d310) desempenho (65,6%) e comer (d550) capacidade (65,6%). Dentre as 24 categorias selecionadas, 12 contemplaram conjuntamente os qualificadores de Desempenho e Capacidade.

Para as categorias referentes ao capítulo “Aprendizagem e aplicação de conhecimento”, as que foram descritas em maior porcentagem como “há dificuldade” foram: aquisição de linguagem (d132) - desempenho (68,8%) e capacidade (96,9%); aprender por meio de ações com objetos (d131) - capacidade (75%); aquisição de conceitos (d137) - capacidade (62,5%) e concentrar a atenção (d160) - capacidade (59,4%).

No capítulo “Tarefas e demandas gerais”, as três categorias selecionadas foram descritas em maior prevalência como “há dificuldade”, sendo elas: lidar com o estresse e outras demandas psicológicas (d240) - desempenho (59,4%); realizar uma única tarefa (d210) - desempenho (50%) e realizar a rotina diária (d230) - desempenho (50%).

Das cinco categorias selecionadas do capítulo “Comunicação”, as que em maior porcentagem foram descritas como “há dificuldade” foram: fala (d330) - desempenho (62,5%) e capacidade (100%); conversação (d350) - capacidade (75%) e recepção de mensagens orais (d310) - capacidade (53,1%). As categorias descritas em maior porcentagem como “não há dificuldade” foram: produção de mensagens não verbais (d335) - capacidade (59,4%) e expressão vocal sem fala (d331) - capacidade (50%).

No capítulo “Cuidado pessoal” verificou-se um número alto de respostas “Não informado”. As categorias lavar-se (d510), cuidado das partes do corpo (d520) e vestir-se (d540) contemplaram apenas o qualificador de desempenho e todas foram mais frequentemente descritas como “há dificuldade” (34,4%). A categoria comer (d550) foi descrita como “não há dificuldade” em 46,9% no qualificador de desempenho e em 34,4% no qualificador de capacidade.

No capítulo “Áreas principais da vida”, as categorias educação infantil (d815) e educação escolar (d820) também contemplaram apenas o qualificador de desempenho. A categoria educação escolar foi descrita como “não se aplica” em 87,5% dos pacientes. A categoria educação infantil foi descrita como “não há dificuldade” em 37,5% e como “há dificuldade” em 31,3%.

A análise de associação entre as categorias do componente Atividades e Participação da CIF e o sexo dos pacientes não apresentou resultado com significância estatística.

Na análise de associação entre as categorias do componente Atividades e Participação da CIF e a idade dos pacientes, categorizada segundo a mediana, houve resultado com significância estatística entre a idade e a categoria resolver problemas (d175) - desempenho, com valor-p de 0,045. Entre os participantes que apresentaram dificuldade, 71,4% tinham idade ≤ 37,5 meses. A análise de associação entre as categorias da CIF e a variável “Frequenta escola” (sim e não) demonstrou resultado com significância estatística com a categoria educação infantil (d815) - desempenho, com valor-p de 0,003, sendo que entre os pacientes que não apresentaram dificuldade, todos frequentam a escola (Tabela 1).

**Tabela 1:** Análise de associação entre as variáveis selecionadas da CIF e os dados sociodemográficos dos participantes

Capítulo 1 - Aprendizagem e aplicação de conhecimento												
	**Desempenho**			**Capacidade**			**Desempenho**			**Capacidade**		
	**Idade (em meses)**			**Idade (em meses)**			**Frequenta Escola**			**Frequenta Escola**		
**Observar (d110)**	≤37,5	>37,5	Total	≤37,5	>37,5	Total	Sim	Não	Total	Sim	Não	Total
	**N (%)**	**N (%)**		**N (%)**	**N (%)**		**N (%)**	**N (%)**		**N (%)**	**N (%)**	
Não há dificuldade	9 (60,0)	6 (40,0)	15 (100,0)	-	-	-	8 (61,5)	5 (38,5)	13 (100,0)	-	-	-
Há dificuldade	1 (25,0)	3 (75,0)	4 (100,0)	-	-	-	3 (100,0)	0 (0,0)	3 (100,0)	-	-	-
Valor-p	0,249			-			0,295			-		
**Ouvir (d115)**	≤37,5	>37,5	Total	≤37,5	>37,5	Total	Sim	Não	Total	Sim	Não	Total
	**N (%)**	**N (%)**		**N (%)**	**N (%)**		**N (%)**	**N (%)**		**N (%)**	**N (%)**	
Não há dificuldade	9 (56,2)	7 (43,8)	16 (100,0)	8 (50,0)	8 (50,0)	16 (100,0)	8 (57,1)	6 (42,9)	15 (100,0)	10 (66,7)	5 (33,3)	15 (100,00)
Há dificuldade	1 (100,0)	0 (0,0)	1 (100,0)	2 (40,0)	3 (60,0)	5 (100,0)	1 (100,0)	0 (0,0)	1 (100,0)	4 (80,0)	1 (20,0)	5 (100,0)
Valor-p	0,588			0,550			0,600			0,517		
**Imitar (d130)**	≤37,5	>37,5	Total	≤37,5	>37,5	Total	Sim	Não	Total	Sim	Não	Total
	**N (%)**	**N (%)**		**N (%)**	**N (%)**		**N (%)**	**N (%)**		**N (%)**	**N (%)**	
Não há dificuldade	-	-	-	5 (55,6)	4 (44,4)	9 (100,0)	-	-	-	8 (100,0)	0 (0,0)	8 (100,0)
Há dificuldade	-	-	-	6 (75,0)	2 (25,0)	8 (100,0)	-	-	-	3 (50,0)	3 (50,0)	6 (100,0)
Valor-p	-			0,373			-			0,055		
**Aprender por meio de ações com objetos (d131)**	≤37,5	>37,5	Total	≤37,5	>37,5	Total	Sim	Não	Total	Sim	Não	Total
	**N (%)**	**N (%)**		**N (%)**	**N (%)**		**N (%)**	**N (%)**		**N (%)**	**N (%)**	
Não há dificuldade	-	-	-	4 (66,7)	2 (33,3)	6 (100,0)	-	-	-	3 (60,0)	2 (40,0)	5 (100,0)
Há dificuldade	-	-	-	12 (50,0)	12 (50,0)	24 (100,0	-	-	-	16 (76,2)	5 (23,8)	23 (100,0)
Valor-p	-			0,395			-			0,411		
**Aquisição de linguagem (d132)**	≤37,5	>37,5	Total	≤37,5	>37,5	Total	Sim	Não	Total	Sim	Não	Total
	**N (%)**	**N (%)**		**N (%)**	**N (%)**		**N (%)**	**N (%)**		**N (%)**	**N (%)**	
Não há dificuldade	1 (33,3)	2 (66,7)	3 (100,0)	0 (0,0)	1 (100,0)	1 (100,0)	2 (100,0)	0 (0,0)	2 (100,0)	0 (0,0)	1 (100,0)	1 (100,0)
Há dificuldade	10 (45,5)	12 (54,5)	22 (100,0)	16 (51,6)	15 (48,4)	31 (100,0)	16 (76,2)	5 (23,8)	23 (100,0)	21(77,8)	6 (22,2)	27 (100,0)
Valor-p	0,593			0,500			0,605			0,250		
**Aquisição de conceitos (d137)**	≤37,5	>37,5	Total	≤37,5	>37,5	Total	Sim	Não	Total	Sim	Não	Total
	**N (%)**	**N (%)**		**N (%)**	**N (%)**		**N (%)**	**N (%)**		**N (%)**	**N (%)**	
Não há dificuldade	-	-	-	0 (0,0)	4 (100,0)	4 (100,0)	-	-	-	4 (100,0)	0 (0,0)	4 (100,0)
Há dificuldade	-	-	-	10 (50,0)	10 (50,0)	20 (100,0)	-	-	-	12 (66,7)	6(33,3)	18 (100,0)
Valor-p	-			0,094			-			0,249		
**Aquisição de habilidade (d155)**	≤37,5	>37,5	Total	≤37,5	>37,5	Total	Sim	Não	Total	Sim	Não	Total
	**N (%)**	**N (%)**		**N (%)**	**N (%)**		**N (%)**	**N (%)**		**N (%)**	**N (%)**	
Não há dificuldade	9 (69,2)	4 (30,8)	13 (100,0)	4 (57,1)	3 (42,9)	7 (100,0)	7 (58,3)	5 (41,7)	12 (100,0)	4 (66,7)	2 (33,3)	6 (100,0)
Há dificuldade	4 (50,0)	4 (50,0)	8 (100,0)	2 (28,6)	5 (71,4)	7 (100,0)	6 (75,0)	2 (25,0)	8 (100,0)	6 (85,7)	1(14,3)	7 (100,0)
Valor-p	0,336			0,296			0,392			0,437		
**Concentrar a atenção (d160)**	≤37,5	>37,5	Total	≤37,5	>37,5	Total	Sim	Não	Total	Sim	Não	Total
	**N (%)**	**N (%)**		**N (%)**	**N (%)**		**N (%)**	**N (%)**		**N (%)**	**N (%)**	
Não há dificuldade	4 (66,7)	2 (33,3)	6 (100,0)	3 (60,0)	2 (40,0)	5 (100,0)	3 (60,0)	2 (40,0)	5 (100,0)	4 (100,0)	0 (0,0)	4 (100,0)
Há dificuldade	4 (40,0)	6 (60,0)	10 (100,0)	10 (52,6)	9 (47,4)	19 (100,0)	8 (80,0)	2 (20,0)	10 (100,0)	12 (70,6)	5 (29,4)	17 (100,0)
Valor-p	0,304			0,585			0,407			0,304		
**Resolver problemas (d175)**	≤37,5	>37,5	Total	≤37,5	>37,5	Total	Sim	Não	Total	Sim	Não	Total
	**N (%)**	**N (%)**		**N (%)**	**N (%)**		**N (%)**	**N (%)**		**N (%)**	**N (%)**	
Não há dificuldade	0 (0,0)	4 (100,0)	4 (100,0)	2 (66,7)	1 (33,3)	3 (100,0)	3 (75,0)	1 (25,0)	4 (100,0)	2 (66,7)	1 (33,3)	3 (100,0)
Há dificuldade	5 (71,4)	2 (28,6)	7 (100,0)	1 (100,0)	0 (0,0)	1 (100,0)	3 (50,0)	3 (50,0)	6 (100,0)	1 (100,0)	0 (0,0)	1 (100,0)
Valor-p	**0,045**			0,750			0,452			0,750		
**Tomar decisões (d177)**	≤37,5	>37,5	Total	≤37,5	>37,5	Total	Sim	Não	Total	Sim	Não	Total
	**N (%)**	**N (%)**		**N (%)**	**N (%)**		**N (%)**	**N (%)**		**N (%)**	**N (%)**	
Não há dificuldade	7 (63,6)	4 (36,4)	11 (100,0)	2 (66,7)	1 (33,3)	3 (100,0)	8 (80,0)	2 (20,0)	10 (100,0)	2 (66,7)	1 (33,3)	3 (100,0)
Há dificuldade	2 (100,0)	0 (0,0)	2 (100,0)	-	-	-	0 (0,0)	2 (100,0)	2 (100,0)	-	-	-
Valor-p	0,462			-			0,091			-		
**Capítulo 2 - Tarefas e demandas gerais**												
	**Desempenho**			**Capacidade**			**Desempenho**			**Capacidade**		
	**Idade (em meses)**			**Idade (em meses)**			**Frequenta Escola**			**Frequenta Escola**		
**Realizar uma única tarefa (d210)**	≤37,5	>37,5	Total	≤37,5	>37,5	Total	Sim	Não	Total	Sim	Não	Total
	**N (%)**	**N (%)**		**N (%)**	**N (%)**		**N (%)**	**N (%)**		**N (%)**	**N (%)**	
Não há dificuldade	5 (33,3)	10 (66,7)	15 (100,0)	15 (55,6)	12 (44,4)	27 (100,0)	11 (84,8)	2 (15,4)	13 (100,0)	16 (69,6)	7 (30,4)	23 (100,0)
Há dificuldade	10 (62,5)	6 (37,5)	16 (100,0)	1 (20,0)	4 (80,0)	5 (100,0)	10 (66,7)	5 (33,3)	15 (100,0)	5 (100,0)	0 (0,0)	5 (100,0)
Valor-p	0,103			0,166			0,258			0,207		
**Realizar uma rotina diária (d230)**	≤37,5	>37,5	Total	≤37,5	>37,5	Total	Sim	Não	Total	Sim	Não	Total
	**N (%)**	**N (%)**		**N (%)**	**N (%)**		**N (%)**	**N (%)**		**N (%)**	**N (%)**	
Não há dificuldade	1 (25,0)	3 (75,0)	4 (100,0)	-	-	-	2 (66,7)	1 (33,3)	3 (100,0)	-	-	-
Há dificuldade	10 (62,5)	6 (37,5)	16 (100,0)	-	-	-	11 (68,8)	5 (31,30	16 (100,0)	-	-	-
Valor-p	0,217			-			0,705			-		
**Lidar com o estresse e outras demandas psicológicas (d240)**	≤37,5	>37,5	Total	≤37,5	>37,5	Total	Sim	Não	Total	Sim	Não	Total
	**N (%)**	**N (%)**		**N (%)**	**N (%)**		**N (%)**	**N (%)**		**N (%)**	**N (%)**	
Não há dificuldade	6 (46,2)	7 (53,8)	13 (100,0)	-	-	-	-	-	-	-	-	-
Há dificuldade	-	-	-	-	-	-	9 (75,0)	3 (25,0)	12 (100,0)	-	-	-
Valor-p	-			-			-			-		
**Capítulo 3 - Comunicação**												
	**Desempenho**			**Capacidade**			**Desempenho**			**Capacidade**		
	**Idade (em meses)**			**Idade (em meses)**			**Frequenta Escola**			**Frequenta Escola**		
**Recepção de mensagens orais (d310)**	≤37,5	>37,5	Total	≤37,5	>37,5	Total	Sim	Não	Total	Sim	Não	Total
	**N (%)**	**N (%)**		**N (%)**	**N (%)**		**N (%)**	**N (%)**		**N (%)**	**N (%)**	
Não há dificuldade	5 (55,6)	4 (44,4)	9 (100,0)	5 (41,7)	7 (58,3)	12 (100,0)	6 (75,0)	2 (25,0)	8 (100,0)	8 (80,0)	2 (20,0)	10 (100,0)
Há dificuldade	2 (100,0)	0 (0,0)	2 (100,0)	10 (58,8)	7 (41,2)	17 (100,0)	2 (100,0)	0 (0,0)	2 (100,0)	11 (73,3)	4 (26,7)	15 (100,0)
Valor-p	0,382			0,297			0,622			0,545		
**Fala (d330)**	≤37,5	>37,5	Total	≤37,5	>37,5	Total	Sim	Não	Total	Sim	Não	Total
	**N (%)**	**N (%)**		**N (%)**	**N (%)**		**N (%)**	**N (%)**		**N (%)**	**N (%)**	
Não há dificuldade	-	-	-	-	-	-	-	-	-	-	-	-
Há dificuldade	11 (55,0)	9 (45,0)	20 (100,0)	16 (50,0)	16 (50,0)	32 (100,0)	14 (70,0)	6 (30,0)	20 (100,0)	21 (75,0)	7 (25,0)	28 (100,0)
Valor-p	-			-			-			-		
**Expressão vocal sem fala (d331)**	≤37,5	>37,5	Total	≤37,5	>37,5	Total	Sim	Não	Total	Sim	Não	Total
	**N (%)**	**N (%)**		**N (%)**	**N (%)**		**N (%)**	**N (%)**		**N (%)**	**N (%)**	
Não há dificuldade	2 (100,0)	-	2 (100,0)	7 (43,8)	9 (56,2)	16 (100,0)	1 (100,0)	0 (0,0)	1 (100,0)	11 (84,6)	2 (15,4)	13 (100,0)
Há dificuldade	-	-	-	1 (100,0)	0 (0,0)	1 (100,0)	-	-	-	-	-	-
Valor-p	-			0,471			-			-		
**Produção de mensagens não verbais (d335)**	≤37,5	>37,5	Total	≤37,5	>37,5	Total	Sim	Não	Total	Sim	Não	Total
	**N (%)**	**N (%)**		**N (%)**	**N (%)**		**N (%)**	**N (%)**		**N (%)**	**N (%)**	
Não há dificuldade	7 (70,0)	3 (30,0)	10 (100,0)	13 (68,4)	6 (31,6)	19 (100,0)	6 (66,7)	3 (33,3)	9 (100,0)	12 (75,0)	4 (25,0)	16 (100,0)
Há dificuldade	1 (100,0)	0 (0,0)	1 (100,0)	0 (0,0)	1 (100,0)	1 (100,0)	1 (100,0)	0 (0,0)	1 (100,0)	1 (100,0)	0 (0,0)	1 (100,0)
Valor-p	0,727			0,350			0,700			0,765		
**Conversação (d350)**	≤37,5	>37,5	Total	≤37,5	>37,5	Total	Sim	Não	Total	Sim	Não	Total
	**N (%)**	**N (%)**		**N (%)**	**N (%)**		**N (%)**	**N (%)**		**N (%)**	**N (%)**	
Não há dificuldade	-	-	-	3 (50,0)	3 (50,0)	6 (100,0)	-	-	-	3 (50,0)	3 (50,0)	6 (100,0)
Há dificuldade	-	-	-	13 (54,2)	11 (45,8)	-	-	-	-	16 (80,0)	4 (20,0)	20 (100,0)
Valor-p	-			0,605			-			0,175		
**Capítulo 5 - Cuidado pessoal**												
	**Desempenho**			**Capacidade**			**Desempenho**			**Capacidade**		
	**Idade (em meses)**			**Idade (em meses)**			**Frequenta Escola**			**Frequenta Escola**		
**Lavar-se (d510)**	≤37,5	>37,5	Total	≤37,5	>37,5	Total	Sim	Não	Total	Sim	Não	Total
	**N (%)**	**N (%)**		**N (%)**	**N (%)**		**N (%)**	**N (%)**		**N (%)**	**N (%)**	
Não há dificuldade	2 (66,7)	1 (33,3)	3 (100,0)	-	-	-	1 (50,0)	1 (50,0)	2 (100,0)	-	-	-
Há dificuldade	7 (63,6)	4 (36,4)	11 (100,0)	-	-	-	7 (63,6)	4 (36,4)	11 (100,0)	-	-	-
Valor-p	0,725			-			0,641			-		
**Cuidados das partes do corpo (d520)**	≤37,5	>37,5	Total	≤37,5	>37,5	Total	Sim	Não	Total	Sim	Não	Total
	**N (%)**	**N (%)**		**N (%)**	**N (%)**		**N (%)**	**N (%)**		**N (%)**	**N (%)**	
Não há dificuldade	1 (33,3)	2 (66,7)	3 (100,0)	-	-	-	1 (50,0)	1 (50,0)	2 (100,0)	-	-	-
Há dificuldade	7 (63,6)	4 (36,4)	11 (100,0)	-	-	-	8 (72,7)	3 (27,3)	11 (100,0)	-	-	-
Valor-p	0,385			-			0,538			-		
**Vestir-se (d540)**	≤37,5	>37,5	Total	≤37,5	>37,5	Total	Sim	Não	Total	Sim	Não	Total
	**N (%)**	**N (%)**		**N (%)**	**N (%)**		**N (%)**	**N (%)**		**N (%)**	**N (%)**	
Não há dificuldade	0 (0,0)	2 (100,0)	2 (100,0)	-	-	-	1 (50,0)	1 (50,0)	2 (100,0)	-	-	-
Há dificuldade	7 (63,6)	4 (36,4)	11 (100,0)	-	-	-	7 (63,6)	4 (36,4)	11 (100,0)	-	-	-
Valor-p	0,192			-			0,641			-		
**Comer (d550)**	≤37,5	>37,5	Total	≤37,5	>37,5	Total	Sim	Não	Total	Sim	Não	Total
	**N (%)**	**N (%)**		**N (%)**	**N (%)**		**N (%)**	**N (%)**		**N (%)**	**N (%)**	
Não há dificuldade	10 (66,7)	5 (33,3)	15 (100,0)	8 (72,7)	3 (27,3)	11 (100,0)	8 (53,3)	7 (46,7)	15 (100,0)	5 (50,0)	5 (50,0)	10 (100,0)
Há dificuldade	2 (50,0)	2 (50,0)	4 (100,0)	-	-	-	3 (100,0)	0 (0,0)	3 (100,0)	-	-	-
Valor-p	0,475			-			0,202			-		
**Capítulo 8 - Áreas principais da vida**												
	**Desempenho**			**Capacidade**			**Desempenho**			**Capacidade**		
	**Idade (em meses)**			**Idade (em meses)**			**Frequenta Escola**			**Frequenta Escola**		
**Educação infantil (d815)**	≤37,5	>37,5	Total	≤37,5	>37,5	Total	Sim	Não	Total	Sim	Não	Total
	**N (%)**	**N (%)**		**N (%)**	**N (%)**		**N (%)**	**N (%)**		**N (%)**	**N (%)**	
Não há dificuldade	5 (41,7)	7 (58,3)	12 (100,0)	-	-	-	12 (100,0)	0 (0,0)	12 (100,0)	-	-	-
Há dificuldade	7 (70,0)	3 (30,0)	10 (100,0)	-	-	-	4 (40,0)	6 (60,0)	10 (100,0	-	-	-
Valor-p	0,185			-			**0,003**			-		
**Educação escolar (d820)**	≤37,5	>37,5	Total	≤37,5	>37,5	Total	Sim	Não	Total	Sim	Não	Total
	**N (%)**	**N (%)**		**N (%)**	**N (%)**		**N (%)**	**N (%)**		**N (%)**	**N (%)**	
Não há dificuldade	-	1 (100,0)	1 (100,0)	-	-	-	1 (100,0)	-	1 (100,0)	-	-	-
Há dificuldade	-	3 (100,0)	3 (100,0)	-	-	-	3 (100,0)	-	3 (100,0)	-	-	-
Valor-p	-			-			-			-		

N=número de participantes, as informações variam de acordo com as características das informações ou dados faltantes; Teste exato de Fisher

Quanto a associação entre as categorias da CIF e a variável “Diagnóstico Fonoaudiológico” (transtorno do desenvolvimento de linguagem e transtorno de linguagem associado a outras condições) houve significância estatística entre o diagnóstico fonoaudiológico e a categoria conversação (d350) - capacidade (p=0,013), em que entre os participantes que apresentaram dificuldade, 77,3% apresentaram como diagnóstico fonoaudiológico Transtorno de Linguagem associado a outras condições (Tabela 2).

**Tabela 2:** Análise de associação entre variáveis selecionadas da CIF e o diagnóstico fonoaudiológico

**Capítulo 1 - Aprendizagem e aplicação de conhecimento**						
	**Desempenho**			**Capacidade**		
	Diagnóstico Fonoaudiológico			Diagnóstico Fonoaudiológico		
**Observar (d110)**	TDL**	TL***	Total	TDL**	TL***	Total
	**N (%)**	**N (%)**		**N (%)**	**N (%)**	
Não há dificuldade	6 (42,9)	8 (57,1)	14,0	-	-	-
Há dificuldade	0 (0,0)	4 (100,0)	4 (100,0)	-	-	-
Valor-p	0,162			-		
**Ouvir (d115)**	TDL**	TL***	Total	TDL**	TL***	Total
	**N (%)**	**N (%)**		**N (%)**	**N (%)**	
Não há dificuldade	3 (20,0)	12 (80,0)	15 (100,0)	7 (46,7)	8 (53,3)	15 (100,0)
Há dificuldade	1 (100,0)	0 (0,0)	1 (100,0)	2 (40,0)	3 (60,0)	5 (100,0)
Valor-p	0,250			0,604		
**Imitar (d130)**	TDL**	TL***	Total	TDL**	TL***	Total
	**N (%)**	**N (%)**		**N (%)**	**N (%)**	
Não há dificuldade	-	-	-	5 (55,6)	4 (44,4)	9 (100,0)
Há dificuldade	-	-	-	1 (14,3)	6 (85,7)	7 (100,0)
Valor-p	-			0,121		
**Aprender por meio de ações com objetos (d131)**	TDL**	TL***	Total	TDL**	TL***	Total
	**N (%)**	**N (%)**		**N (%)**	**N (%)**	
Não há dificuldade	-	-	-	2 (33,3)	4 (66,7)	6 (100,0)
Há dificuldade	-	-	-	9 (40,9)	13 (59,1)	22 (100,0)
Valor-p	-			0,561		
**Aquisição de linguagem (d132)**	TDL**	TL***	Total	TDL**	TL***	Total
	**N (%)**	**N (%)**		**N (%)**	**N (%)**	
Não há dificuldade	3 (100,0)	0 (0,0)	3 (100,0)	1 (100,0)	0 (0,0)	1 (100,0)
Há dificuldade	7 (35,0)	13 (65,0)	20 (100,0)	11 (37,9)	18 (62,1)	29 (100,0)
Valor-p	0,068			0,400		
**Aquisição de conceitos (d137)**	TDL**	TL***	Total	TDL**	TL***	Total
	**N (%)**	**N (%)**		**N (%)**	**N (%)**	
Não há dificuldade	-	-	-	2 (50,0)	2 (50,0)	4 (100,0)
Há dificuldade	-	-	-	7 (36,8)	12 (63,2)	19 (100,0)
Valor-p	-			0,517		
**Aquisição de habilidade (d155)**	TDL**	TL***	Total	TDL**	TL***	Total
	**N (%)**	**N (%)**		**N (%)**	**N (%)**	
Não há dificuldade	7 (58,3)	5 (41,7)	12 (100,0)	3 (42,9)	4 (57,1)	7 (100,0)
Há dificuldade	3 (37,5)	5 (62,5)	8 (100,0)	4 (57,1)	3 (42,9)	7 (100,0)
Valor-p	0,325			0,500		
**Concentrar a atenção (d160)**	TDL**	TL***	Total	TDL**	TL***	Total
	**N (%)**	**N (%)**		**N (%)**	**N (%)**	
Não há dificuldade	3 (60,0)	2 (40,0)	5 (100,0)	3 (60,0)	2 (40,0)	5 (100,0)
Há dificuldade	5 (50,0)	5 (50,0)	10 (100,0)	5 (27,8)	13 (72,2)	18 (100,0)
Valor-p	0,573			0,208		
**Resolver problemas (d175)**	TDL**	TL***	Total	TDL**	TL***	Total
	**N (%)**	**N (%)**		**N (%)**	**N (%)**	
Não há dificuldade	2 (50,0)	2 (50,0)	4 (100,0)	0 (0,0)	3 (100,0)	3 (100,0)
Há dificuldade	2 (33,3)	4 (66,7)	6 (100,0)	1 (100,0)	0 (0,0)	1 (100,0)
Valor-p	0,548			0,250		
**Tomar decisões (d177)**	TDL**	TL***	Total	TDL**	TL***	Total
	**N (%)**	**N (%)**		**N (%)**	**N (%)**	
Não há dificuldade	6 (54,5)	5 (45,5)	11 (100,0)	1 (33,3)	2 (66,7)	3 (100,0)
Há dificuldade	0 (0,0)	1 (100,0)	1 (100,0)	-	-	-
Valor-p	0,500			-		
**Capítulo 2 - Tarefas e demandas gerais**						
	**Desempenho**			**Capacidade**		
	Diagnóstico Fonoaudiológico			Diagnóstico Fonoaudiológico		
**Realizar uma única tarefa (d210)**	TDL**	TL***	Total	TDL**	TL***	Total
	**N (%)**	**N (%)**		**N (%)**	**N (%)**	
Não há dificuldade	6 (42,9)	8 (57,1)	14 (100,0)	9 (36,0)	16 (64,0)	25 (100,0)
Há dificuldade	6 (40,0)	9 (60,0)	15 (100,0)	3 (60,0)	2 (40,0)	5 (100,0)
Valor-p	0,587			0,304		
**Realizar uma rotina diária (d230)**	TDL**	TL***	Total	TDL**	TL***	Total
	**N (%)**	**N (%)**		**N (%)**	**N (%)**	
Não há dificuldade	2 (50,0)	2 (50,0)	4 (100,0)	-	-	-
Há dificuldade	7 (46,7)	8 (53,3)	15 (100,0)	-	-	-
Valor-p	0,667			-		
**Lidar com o estresse e outras demandas psicológicas (d240)**	TDL**	TL***	Total	TDL**	TL***	Total
	**N (%)**	**N (%)**		**N (%)**	**N (%)**	
Não há dificuldade	-	-	-	-	-	-
Há dificuldade	6 (50,0)	6 (50,0)	12 (100,0)	-	-	-
Valor-p	-			-		
**Capítulo 3 - Comunicação**						
	**Desempenho**			**Capacidade**		
	Diagnóstico Fonoaudiológico			Diagnóstico Fonoaudiológico		
**Recepção de mensagens orais (d310)**	TDL**	TL***	Total	TDL**	TL***	Total
	**N (%)**	**N (%)**		**N (%)**	**N (%)**	
Não há dificuldade	4 (50,0)	4 (50,0)	8 (100,0)	4 (36,4)	7 (63,6)	11 (100,0)
Há dificuldade	1 (50,0)	1 (50,0)	2 (100,0)	7 (43,8)	9 (56,3)	16 (100,0)
Valor-p	0,778			0,508		
**Fala (d330)**	TDL**	TL***	Total	TDL**	TL***	Total
	**N (%)**	**N (%)**		**N (%)**	**N (%)**	
Não há dificuldade	-	-	-	-	-	-
Há dificuldade	8 (44,4)	10 (55,6)	18 (100,0)	12 (40,0)	18 (60,0)	30 (100,0)
Valor-p	-			-		
**Expressão vocal sem fala (d331)**	TDL**	TL***	Total	TDL**	TL***	Total
	**N (%)**	**N (%)**		**N (%)**	**N (%)**	
Não há dificuldade	0 (0,0)	2 (100,0)	2 (100,0)	3 (20,0)	12 (80,0)	15 (100,0)
Há dificuldade	-	-	-	0 (0,0)	1 (100,0)	1 (100,0)
Valor-p	-			0,813		
**Produção de mensagens não verbais (d335)**	TDL**	TL***	Total	TDL**	TL***	Total
	**N (%)**	**N (%)**		**N (%)**	**N (%)**	
Não há dificuldade	3 (30,0)	7 (70,0)	10 (100,0)	7 (38,9)	11 (61,1)	18 (100,0)
Há dificuldade	0 (0,0)	1 (100,0)	1 (100,0)	0 (0,0)	1 (100,0)	1 (100,0)
Valor-p	0,727			0,632		
**Conversação (d350)**	TDL**	TL***	Total	TDL**	TL***	Total
	**N (%)**	**N (%)**		**N (%)**	**N (%)**	
Não há dificuldade	-	-	-	5 (83,3)	1 (16,7)	6 (100,0)
Há dificuldade	-	-	-	5 (22,7)	17 (77,3)	22 (100,0)
Valor-p	-			**0,013**		
**Capítulo 5 - Cuidado pessoal**						
	**Desempenho**			**Capacidade**		
	Diagnóstico Fonoaudiológico			Diagnóstico Fonoaudiológico		
**Lavar-se (d510)**	TDL**	TL***	Total	TDL**	TL***	Total
	**N (%)**	**N (%)**		**N (%)**	**N (%)**	
Não há dificuldade	3 (100,0)	0 (0,0)	3 (100,0)	-	-	-
Há dificuldade	5 (50,0)	5 (50,0)	10 (100,0)	-	-	-
Valor-p	0,196			-		
**Cuidados das partes do corpo (d520)**	TDL**	TL***	Total	TDL**	TL***	Total
	**N (%)**	**N (%)**		**N (%)**	**N (%)**	
Não há dificuldade	3 (100,0)	0 (0,0)	3 (100,0)	-	-	-
Há dificuldade	6 (54,5)	5 (45,5)	11 (100,0)	-	-	-
Valor-p	0,231			-		
**Vestir-se (d540)**	TDL**	TL***	Total	TDL**	TL***	Total
	**N (%)**	**N (%)**		**N (%)**	**N (%)**	
Não há dificuldade	2 (100,0)	0 (0,0)	2 (100,0)	-	-	-
Há dificuldade	5 (50,0)	5 (50,0)	10 (100,0)	-	-	-
Valor-p	0,318			-		
**Comer (d550)**	TDL**	TL***	Total	TDL**	TL***	Total
	**N (%)**	**N (%)**		**N (%)**	**N (%)**	
Não há dificuldade	8 (57,1)	6 (42,9)	14 (100,0)	5 (50,0)	5 (50,0)	10 (100,0)
Há dificuldade	0 (0,0)	4 (100,0)	4 (100,0)	-	-	-
Valor-p	0,069			-		
**Capítulo 8 - Áreas principais da vida**						
	**Desempenho**			**Capacidade**		
	Diagnóstico Fonoaudiológico			Diagnóstico Fonoaudiológico		
**Educação infantil (d815)**	TDL**	TL***	Total	TDL**	TL***	Total
	**N (%)**	**N (%)**		**N (%)**	**N (%)**	
Não há dificuldade	5 (45,5)	6 (54,5)	11 (100,0)	-	-	-
Há dificuldade	3 (30,0)	7 (70,0)	10 (100,0)	-	-	-
Valor-p	0,392			-		
**Educação escolar (d820)**	TDL**	TL***	Total	TDL**	TL***	Total
	**N (%)**	**N (%)**		**N (%)**	**N (%)**	
Não há dificuldade	0 (0,0)	1 (100,0)	1 (100,0)	-	-	-
Há dificuldade	3 (100,0)	0 (0,0)	3 (100,0)	-	-	-
Valor-p	0,250			-		

N=número de participantes, as informações variam de acordo com as características das informações ou dados faltantes;

** Transtorno de Desenvolvimento de Linguagem;

*** Transtorno de Linguagem associado a outras condições; Teste exato de Fisher

## DISCUSSÃO

A diversidade de categorias do componente Atividades e Participação da CIF encontradas no presente estudo, permitiu a reflexão sobre os impactos que os transtornos de linguagem oral podem representar ao desenvolvimento infantil, principalmente com relação a limitações de atividades e restrições de participação. Deste modo, o estudo buscou identificar as categorias referentes ao componente Atividades e Participação da CIF, e suas associações com idade, sexo, escolaridade e diagnóstico fonoaudiológico global, no contexto de um ambulatório de linguagem infantil.

Quanto aos dados sociodemográficos, o presente estudo revela que a maioria dos participantes pertence ao sexo masculino, resultado similar a outros estudos, que associam a prevalência de alterações de linguagem infantil para o sexo masculino devido a fatores neurológicos, hormonais e sociais^(14-19)^. Quanto aos achados na amostra referentes a idade e escolaridade, em estudo sobre a prevalência das alterações fonoaudiológicas em crianças, foi observado a prevalência das alterações comunicativas na idade pré-escolar (48,7% dos participantes de 36 a 72 meses)^(16)^. Vale destacar ainda que o presente estudo apresenta uma faixa etária ampla, 14 a 96 meses, o que configura uma amplitude bem maior para se pensar e referir às etapas do desenvolvimento e alterações comunicativas.

A predominância do diagnóstico fonoaudiológico de Transtorno de linguagem associado a outras condições corrobora estudo sobre o perfil diagnóstico de um ambulatório de Fonoaudiologia na área de Linguagem infantil, em que foi verificado uma alta prevalência de comorbidades (76,9%), sendo que as doenças neurológicas e o atraso do desenvolvimento neuropsicomotor foram as associações de maior ocorrência^(17)^.

Em relação às categorias do componente Atividades e Participação da CIF, os resultados demonstram que, a pré-seleção dos capítulos e categorias que se relacionam às alterações de linguagem oral infantil favorece sua aplicação, uma vez que todos os capítulos e categorias selecionados foram contemplados no presente estudo. A importância da consideração dos fatores associados às limitações de atividades e restrições de participação é refletida na multiplicidade de categorias também encontrada em outros estudos^(20-23)^, que evidencia a diversidade de manifestações fonoaudiológicas presentes em um mesmo diagnóstico de linguagem. Em estudo sobre as expectativas e os resultados observados após a terapia de fala e linguagem de crianças em idade pré-escolar, foi verificado que a maioria das preocupações, expectativas e evoluções observadas pelos pais e médicos estavam relacionadas a limitações de atividades^(21)^, o que demonstra a importância da integração dos aspectos da funcionalidade, principalmente aqueles relacionados às limitações de atividades e restrições de participação, na atuação fonoaudiológica dos transtornos de linguagem oral infantil.

Em estudo sobre a caracterização do desempenho, em aspectos fonoaudiológicos, de pacientes ambulatoriais segundo a versão da CIF para Crianças e Jovens (CIF-CJ), foi verificada a prevalência da descrição das dificuldades nas categorias relacionadas com questões de linguagem, aprendizagem ou escolares^(20)^. O estudo referiu com maior ocorrência a descrição de dificuldade nas categorias pertencentes aos capítulos de aprendizado básico e aplicação de conhecimentos, sendo elas: aprender a ler (d140) - desempenho, dirigir a atenção (d161) - desempenho, escrever (d170) - capacidade e aprender a escrever (d145) - capacidade. Os estudos se coincidem quanto a prevalência da descrição da dificuldade no capítulo “Aprendizagem e aplicação de conhecimento”, no entanto, se diferem quanto às categorias descritas em maior porcentagem como “há dificuldade”. Tal diferença pode ser justificada pelo cenário, caracterização da amostra, faixa etária e alterações apresentadas pelos pacientes de cada estudo. O estudo referenciado foi realizado com base na análise de prontuários de pacientes atendidos em um ambulatório de avaliação, com idades entre 5 e 16 anos, em que as alterações poderiam não estar relacionadas à linguagem oral, além da possibilidade de os pacientes apresentarem outros tipos de alterações, como de leitura e escrita. Além disso, com relação à caracterização da amostra, o estudo apresentou como critérios de exclusão pacientes com suspeita ou confirmação dos diagnósticos de Transtornos Globais do Desenvolvimento (TGD).

Por outro lado, em estudo, que realizou a identificação das categorias da CIF em quadros de Transtornos de Linguagem e de Fala, as categorias mais frequentes do componente Atividades e Participação - comunicação-recepção de mensagens orais (d310), fala (d330), aquisição de habilidades (d155) e conversação (d350) - estiveram relacionadas aos capítulos de aprendizagem básica e de comunicação^(22)^, o que se assemelha aos resultados do presente estudo quanto aos capítulos em que a descrição da dificuldade ocorreu com maior frequência e quanto às categorias fala (d330) e conversação (d350,) que também foram umas das descritas com maior porcentagem como “há dificuldade”.

Estudos que associam as categorias relevantes às alterações de linguagem oral com os fatores pessoais e diagnósticos clínicos favorecem a compreensão das condições de saúde. As análises realizadas no presente estudo permitiram verificar associação entre a idade e a categoria resolver problemas (d175) - desempenho, em que a maioria dos participantes que apresentaram dificuldade tinha idade ≤ 37,5 meses, o que pode ser justificada pelo desenvolvimento da autonomia, habilidades sociais ou inserção no ambiente escolar, ou a interação de dois ou mais fatores. Assim, independente do diagnóstico de linguagem, os fatores do desenvolvimento têm papel preponderante. Outro fator importante é a experiência escolar, fundamental para as aquisições sociais e educacionais nos primeiros anos escolares^(24)^. A escola favorece a evolução de estágios cognitivos, fortalecendo suas habilidades de enfrentamento das adversidades e incentivando soluções criativas para seus problemas^(24)^.

A associação entre frequentar a escola e a categoria educação infantil (d815) - desempenho, em que todos os participantes que não apresentaram dificuldade frequentam a escola, pode ser justificada pelo fato de as queixas dos pais de crianças em idade pré-escolar ainda não estarem voltadas às questões escolares, que se iniciam na idade escolar. Após a inserção na escola, os pais tendem a ampliar a percepção de queixas relacionadas às dificuldades escolares. Em estudo realizado com familiares de crianças em idade escolar, as principais queixas que motivaram os encaminhamentos à clínica estavam vinculadas a dificuldades de escrita e leitura, enquanto as queixas relacionadas a aspectos psicológicos/comportamentais foram menos frequentes^(25)^.

Em outro estudo, realizado em um ambulatório de avaliação fonoaudiológica, foi verificada associação entre a escolaridade e o Fator denominado “Família/escola”, pertencente ao componente Atividades e Participação da CIF e composto pelas categorias “Relacionamentos familiares - Desempenho” e “Educação escolar - Desempenho”, em que o Fator apresentou maiores pontuações entre os pacientes de maior idade que cursavam o ensino fundamental, explicada pelas maiores demandas escolares apresentadas por indivíduos no ensino fundamental ou mais velhos^(26)^. Embora os estudos se diferenciem quanto às variáveis analisadas, é possível relacionar a idade escolar às maiores demandas e dificuldades escolares observadas no estudo referenciado. Tal fato reflete a importância da atuação fonoaudiológica englobar a linguagem oral e escrita, antes mesmo da entrada no ensino formal, uma vez que o domínio do sistema linguístico em sua modalidade oral e o desenvolvimento das habilidades metacognitivas e metalinguísticas são fundamentais para o aprendizado de leitura e escrita^(27)^.

A associação verificada entre o diagnóstico fonoaudiológico e a categoria d350 - capacidade, em que a maioria dos participantes com dificuldade apresentou como diagnóstico Transtorno de Linguagem associado a outras condições, pode ser explicada pelo perfil dos pacientes atendidos no serviço, uma vez que o hospital ao qual o ambulatório em questão pertence realiza atendimentos de média e alta complexidade, sendo referência no encaminhamento de serviços como neurologia e genética, e recebendo, em sua maioria, pacientes com Transtorno do Espectro Autista (TEA) e Síndrome de Down, condições em que o prejuízo nas habilidades pragmáticas é frequente, o que gera dificuldades de interação, comunicação social e conversação^(28)^.

Por se tratar de uma análise com base em dados secundários, coletados em prontuários, a limitação do estudo envolve a perda de informações, como evidenciado pelo número alto de respostas “Não informado” na análise descritiva das categorias da CIF, que pode ser justificado pelos instrumentos de anamnese e avaliação utilizados no serviço ainda terem pouca associação com os aspectos de funcionalidade descritos na CIF. Isso evidencia a necessidade do desenvolvimento de instrumentos clínicos padronizados com categorias relevantes às alterações de linguagem infantil, que viabilizem sua implementação na prática clínica do serviço e minimizem a perda de informações gerada na análise dos prontuários. Além disso, o estudo apresenta limitações quanto ao tamanho da amostra pesquisada, no entanto, por se tratar de um ambulatório cuja população não é extensa, a amostra foi representativa do perfil do serviço.

Como avanços, o estudo permite a identificação das principais categorias relevantes às alterações de linguagem oral infantil, a compreensão das limitações de atividades e restrições de participação decorrentes dessa condição, assim como a implementação da lista de categorias construída no serviço, viabilizando a aplicação da CIF em sua prática clínica e o desenvolvimento de um estudo longitudinal futuro com a mesma população, ampliando o uso dos qualificadores que especificam o grau de dificuldade para fazer um comparativo pré e pós terapia fonoaudiológica. Além disso, o presente estudo pode motivar fonoaudiólogos de outros serviços que atuam com perfil de pacientes semelhantes à incorporação e operacionalização da CIF no fluxo clínico-assistencial.

Diante dos resultados encontrados, observa-se a importância do uso da CIF como uma ferramenta para a integração dos impactos decorrentes das alterações de linguagem à funcionalidade e a ampliação da abordagem biopsicossocial na clínica fonoaudiológica.

## CONCLUSÃO

Foram selecionadas 24 categorias do componente Atividades e Participação da CIF, das quais 12 contemplaram conjuntamente os qualificadores de Desempenho e Capacidade, em crianças com transtornos de linguagem oral e que se encontram em terapia fonoaudiológica em um serviço ambulatorial da rede pública. A descrição da dificuldade ocorreu com maior frequência nas categorias pertencentes aos capítulos de aprendizagem e aplicação de conhecimento e comunicação, e estiveram relacionadas a atividades como adquirir linguagem, aprender por meio de ações com objetos, falar e conversar.

Além disso, foi possível verificar associações com significância estatística entre os dados sociodemográficos e os diagnósticos fonoaudiológicos com as categorias do componente Atividades e Participação da CIF.
